# Changes in Insulin Resistance with Different Weight Loss Methods in Patients with Type Two Diabetes Mellitus and Hypertension: A Comparative Clinical Trial

**DOI:** 10.3390/jcm15020546

**Published:** 2026-01-09

**Authors:** Kuat Oshakbayev, Aigul Durmanova, Gani Kuttymuratov, Nurzhan Bikhanov, Altay Nabiyev, Timur Suleimenov, Alisher Idrissov, Tomiris Shakhmarova, Zhanel Mirmanova, Saule Rakhimova, Ulan Kozhamkulov, Ainur Akilzhanova

**Affiliations:** 1 Clinical Academic Department for Internal Medicine, University Medical Center, Syganak Str. 46, 010000 Astana, Kazakhstan; 2 Department of Endocrinology, University Medical Center, Syganak Str. 46, 010000 Astana, Kazakhstan; aigul.durmanova@umc.org.kz; 3 Department of Surgery, University Medical Center, Syganak Str. 46, 010000 Astana, Kazakhstan; 4 Department of Family Medicine, Astana Medical University, Street Beibitshilik 49a, 010000 Astana, Kazakhstan; 5 Laboratory of Genomic and Personalized Medicine, Center for Life Sciences, National Laboratory Astana, Nazarbayev University, 53 Kabanbay Batyr Ave, 010000 Astana, Kazakhstansaule.rakhimova@nu.edu.kz (S.R.); akilzhanova@nu.edu.kz (A.A.); 6 Eurasian Society of Personalized Medicine, 10 R. Koshkarbayev Ave, 010000 Astana, Kazakhstan

**Keywords:** insulin resistance, type 2 diabetes mellitus, hypertension, weight loss, semaglutide, bariatric surgery, very-low-calorie diet

## Abstract

**Background:** The comparative effects of pharmacological treatment, bariatric surgery, and diet on insulin resistance (IR) remain unclear. **Aim:** To study the comparative effects of the methods on IR: pharmacologic, bariatric surgery, and very-low-calorie diet (VLCD) in patients with type 2 diabetes mellitus (T2DM) and hypertension. **Methods:** Design: a 90-day prospective, multicenter, comparative clinical trial including 130 adult patients divided into three groups: Drug, Surgery, and VLCD. Endpoints: HOMA-IR; weight loss; and HbA1c, systolic/diastolic blood pressure (SBP/DBP). **Results:** At 90 days, weight loss in the Surgery (−19.8%) and VLCD groups (−17.4%) was significant (*p* < 0.0001), while in the Drug group, the loss was insignificant (−6.5%; *p* = 0.06). SBP/DBP in the Drug group decreased by −9.5% (*p* = 0.0002) and −4.1% (*p* = 0.09), respectively. SBP/DBP in the Surgery group decreased by −13.6% and −10.6%, respectively (*p* < 0.001), and in the VLCD group, by −23.3% and 21.3%, respectively (*p* < 0.0001). HOMA-IR in Drug, Surgery, and VLCD groups decreased by −42.2% (*p* = 0.004), −87.6% (*p* < 0.0001), and −88.7% (*p* < 0.0001), respectively. In the Drug group, HOMA-IR did not reach the normal level. Correlation-regression analysis revealed a direct correlation between weight loss and a decrease in HOMA-IR (r = 0.526; F = 33.2, *p* < 0.0001). HOMA-IR decreases by 65% if weight decreases by 10%; if weight decreases by 25%, then HOMA-IR decreases by 83%. **Conclusions:** HOMA-IR was associated with weight loss: the greater the weight loss, the lower the HOMA-IR. Weight loss leads to a reduction in the need for antidiabetic/antihypertensive drugs in patients with T2DM and hypertension.

## 1. Introduction

To date, there is no consensus on the underlying cause of insulin resistance (IR) in the pathogenesis of internal organ diseases [1,2]. Some authors believe that IR develops first, followed by other syndromes and diseases, such as hyperinsulinemia (HI) or type two diabetes mellitus (T2DM) [3,4]. These authors suggest that IR is a fundamental pathophysiological defect in the development of T2DM and leads to compensatory HI to maintain normal glucose tolerance [5]. These authors believe that hereditary predisposition to IR and obesity, in combination with low physical activity [6], and overnutrition, determine the development of tissue IR and, as a consequence, compensatory HI [7,8].

Numerous studies of IR defined it as a continuum of cardiovascular, endocrinological, nephrological, and hepatological diseases [9,10]. Other studies have shown that abdominal obesity is a cause of IR. Isolated interventions for IR have been used for over 30 years and have shown improvement in metabolic parameters in patients with T2DM, but this has not solved the main problem—curing diabetes itself [1,11]. Authors showed a simultaneous association of IR with markers of glycemic, cardiometabolic, and atherosclerotic risk [12]. Some researchers proved that when the amount of fat in the body decreases, IR disappears [13,14].

Long-term insulin therapy for T2DM sooner or later leads to a decrease in the synthesis of an already relatively insufficient amount of endogenous insulin, which aggravates IR, leading to an increase in the dose of exogenously administered insulin [15,16].

In 1993, G.M. Reaven proposed renaming metabolic syndrome as “syndrome X,” given the uncertainty regarding the primacy of the components of metabolic syndrome [17]. Many authors have shown that if a person is overweight, over time, impaired glucose tolerance develops, followed by the manifestation of T2DM [18,19]. However, the causal relationships between IR and T2DM and other non-communicable chronic diseases remain unclear [20]. T2DM is usually accompanied by overweight, although within the disease, weight loss tends to decrease somewhat over time [21]. Nowadays, it is well known that weight loss improves health and decreases clinical outcomes [22]. It is known that there are several types of weight loss, defined as pharmacologic, surgical, and dietary [11,23,24]. Although almost every method of weight loss has a benefit for IR independently [25,26], it is still debated which of these methods provides the greatest benefit on clinical and lab outcomes. T2DM and IR are still being discussed [27]. The relationship between progressive weight loss and sustained alterations in insulin levels remains uncertain. We have not seen any studies that directly indicate the level of weight loss at which IR and HI can be completely remitted.

What explains the fact that simple targeted weight loss leads to normalization of IR and blood sugar levels in T2DM, and what pathophysiological mechanisms are involved [24,28,29]? What pathophysiological processes occur in cells when they are oversaturated with nutrients? The aim of the study was to study the comparative effects of three main weight loss methods on IR: pharmacological, bariatric surgery, and very-low-calorie diet (VLCD) in patients with T2DM and hypertension.

## 2. Methods

### 2.1. Study Design

A 90-day open-label, prospective, multicenter, comparative clinical trial with the intention-to-treat analysis. Based on the results of a clinical trial and a literature review, a concept for the development of IR in T2DM associated with overweight dysfunction was developed.

### 2.2. Participants

**Figure 1:**
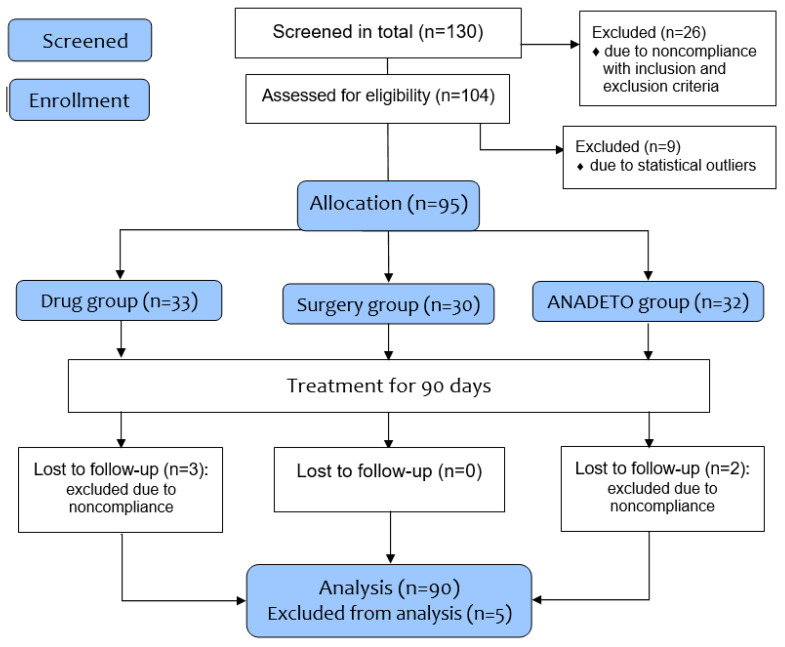
CONSORT 2010 Flow Diagram. Changes in insulin resistance with different weight loss methods in patients with type 2 diabetes mellitus and hypertension: a comparative clinical trial. Trial Registration: ClinicalTrials.gov NCT06410352: https://register.clinicaltrials.gov/prs/app/action/SelectProtocol?sid=S000EG8K&selectaction=Edit&uid=U0006MBT&ts=56&cx=-vph5l9 (5 August 2024).

In total, 130 participants were screened, and 104 adults (63 females) aged 30 to 60 years with T2DM and hypertension were included in the study to determine eligibility; 26 were excluded based on the inclusion/exclusion criteria as described below; nine patients were excluded due to statistical outliers (Figure 1). The remaining 95 patients were unevenly and voluntarily allocated into three groups: Drug (pharmacological); Surgery (bariatric surgery); and VLCD. Of these patients included in the treatment groups, five patients (three from the Drug group and two from the VLCD group) were excluded due to noncompliance and drug intolerance. Finally, 90 patients (86% of 104 patients) were included in the study for analysis (File S1: PROTOCOL trial_T2D_CVD_Eng, a description of the design of this study as part of a larger study, as well as information about its ethical approval and monitoring, File S2: CONSORT Extension_Treatment Checklist, are provided in the Supplementary Materials).

Inclusion criteria: (1) written informed consent; (2) T2DM ≥ 3 years with glucose-lowering therapy including insulin; (3) hypertension ≥ 3 years of treatment; (4) 30–60 years old; (5) BMI ≥ 27 kg/m^2^ for both sexes, for Asian ethnicity; (6) weight loss at baseline. All included patients before recruiting to the study received standard-of-care treatment for T2DM and hypertension [30].

Exclusion criteria: (1) T1DM; (2) <30 or >61 years old; (3) patients after bariatric surgery; (4) unstable cardiac disorders (severe uncontrolled hypertension, uncontrolled arrhythmias, refractory angina, New York Heart Association class IV heart failure, or critical valvular heart disease); (5) glomerular filtration rate < 40 mL/min and/or dialysis within 14 days before screening; (6) ejection fraction < 40%; (7) history of alcohol consumption > 30 g/day within the past 3 years; (8) malignancy within the past 5 years; (9) gestation or lactation; (10) hereditary diseases; (11) known hypersensitivity to any of the test substances.

Outcome measures. Primary endpoints: The Homeostasis Model Assessment of Insulin Resistance Index (HOMA-IR), blood insulin, weight loss, HbA1c, and fasting blood glucose. Secondary endpoints: systolic/diastolic blood pressure (SBP and DBP); lipids.

Patient recruitment and randomization. Patient recruitment was carried out at three different clinical centers (Department for Internal Medicine, Department for Endocrinology, and Department for Surgery). The patients were then consulted by two doctors (a surgeon and a therapist) so that the patient could choose the type of treatment that was right for him/her. Each patient was informed of the pros and cons of each treatment method. After assessing their condition and meeting all indications/contraindications, as well as inclusion/exclusion criteria, they were referred to the appropriate center for the appropriate weight loss treatment. Randomization and blinding of patients in the study were not possible due to its design. The comparative methods involve different interventions; patient allocation cannot be performed ethically; informed consent cannot be concealed; results may not always correspond to the results of the comparative treatments; and randomization requires clinical equipoise [31,32].

The patients were allocated to each group based on equal baseline characteristics according to the inclusion/exclusion criteria, including adjustments for baseline and confounding characteristics. The patients were allocated to each group based on the decision of the patient and two physicians (a surgeon and a therapist) to avoid the risk of “selection bias.” Parameter normalization focused on the work needed to integrate processes into statistics.

The data were collected by three different research teams, who had no access to patient data other than their own. All data (anthropometric, body composition analysis, clinical, laboratory, and instrumental data) were collected in the same place. Laboratory technicians, instrument specialists, and statisticians were blinded to the group(s) to which the patient belonged. The statistician could only work with the data across all study groups once, when all the data had been collected, and the database was locked, and only then could we break the code and see which treatment was best. All tests were performed in the same clinical laboratory, certified and accredited according to international quality management systems. A combination of in-person conversations and telephone calls was conducted during the study period.

Definition of the term “overweight dysfunction.” Lipids in the body perform various functions, such as an energy source, a shock-absorbing cushion for organs, an insulating function, a fat depot, and the adsorption of various substances [33,34].

Excess weight is a component of lipids, which are stored as fat. The primary role of overweight is to serve as an energy source in the absence of available food. Consequently, overweight dysfunction occurs when the body does not utilize fat for a long period of time, which leads to the interference of this fat in the body’s metabolic processes [35].

Interventions (Figure 1). Drug group (*n* = 30) received subcutaneous Semaglutide (GLP-1RA) 1 mg once per 7 day with oral Empagliflozin (SGLT-2i) 25 mg once per day additionally to standard glucose-lowering treatment (metformin, sulfonylureas) including antihypertensive, lipid-lowering, and symptomatic therapy; a typical low-carbohydrate, low-salt balanced eating, including low-carb principles (focuses on whole, unprocessed foods, nutrient-rich, high-fiber foods, drastically cutting refined carbs and sugar while emphasizing non-starchy veggies, healthy fats, and quality lean proteins (meat, fish, eggs), using foods like spinach, avocado, lean proteins, and healthy oils, avoiding sugary drinks, refined carbs, and white flour products, and low-sodium (often <2300 mg/day)) [30], diet with an emphasis on that help regulate blood sugar levels, promote satiety, and minimize gastrointestinal side effects [29].

The Surgery group (*n* = 30) received a laparoscopic minigastric bypass (MGB), which t is an endo-videoscope technique using intraperitoneal synthetic/biological materials to reduce the absorption surface of the gastrointestinal tract by bypassing most of the stomach, duodenum, and initial section of the small intestine, which reduces food absorption and results in decreased production of gastrointestinal hormones [28]. The patients pass through additional pre-operation examinations (blood analysis, ultrasound, electrocardiography, esophagogastroscopy, and other necessary standard methods). Postoperative management of the patients was carried out in accordance with traditional recommendations [28,36].

The VLCD group (*n* = 30) received a weight loss program named “analimentary detoxication” (ANADETO; Astana city, invention patents the Republic of Kazakhstan #RK32138 [37]; Eurasian patent #EA020034) [38]) including <100 kcal/day with fat-free vegetables/greens (tomato/cucumber, dill, parsley, green onions, and lemon 15–20 g/day in various combinations) and salt intake (5–6 g/day), optimal physical activity (>7000 steps per day), and sexual self-restraint [39]. The ANADETO weight loss program was administered twice over 90 days. The program is aimed at the following outputs: (a) use of own fatty store (autolipophagy); (b) control endogen metabolic intoxication; (c) reuse of interim metabolic substrates. During the 90-day treatment, the following regimen was used: the first 30 days—ANADETO; the second 30 days—a 20:4 intermittent fasting protocol [40] to maintain the achieved weight during the first ANADETO; the third 30 days—also ANADETO.

Due to the presence of many known contraindications to the use of the drugs and surgical intervention, the Drug and Surgery groups included patients with a milder clinical and laboratory course of the diseases, but baseline body weight, insulin levels, HOMA-IR, and HbA1c did not differ significantly between the comparison groups [41] (Table 1).

### 2.3. Analytical Assessment

Anthropometrical indicators included age (years), weight (kg), BMI (kg/m^2^), and waist circumference (cm). Body composition parameters, including fat mass (in % of total body weight), fat-free mass, total body water, muscle mass, and bone mass, were measured using a Tanita MC-780MA Body Composition Analyzer (Tanita Corp., Tokyo, Japan).

Physical activity was assessed as the number of steps taken by patients, as determined by individual pedometers from Hoffmann-La Roche, Inc. (Basel, Switzerland) or other individual digital systems.

Laboratory study. On the same blood samples, the standard laboratory performed a complete blood count, erythrocyte sedimentation rate, glucose, HbA1c, creatinine, urea, electrolytes, lipid profile (total cholesterol, HDL/LDL, triglycerides), total proteins, bilirubin, and hepatic enzyme activities. Fasting serum insulin was determined by electrochemiluminescence immunoassay incorporating the “ECLIA” method (Roche diagnostics, GmbH, Cobas^®^, Elecsys Insulin kit, Indianapolis, IN, USA). The value was expressed as nU/L, and hyperinsulinemia was considered >12.5 nU/L.

We used HOMA-IR as a surrogate measure of insulin sensitivity as follows: HOMA-IR = ((fasting insulin in nU/L) × (fasting glucose in mmol/L)/22.5). Early insulin resistance was considered if the index was >2, and significant insulin resistance if >2.9.

Imaging. Ultrasound imaging (GE Vivid 7 Ultrasound; GE Healthcare Worldwide, US, MI, USA) was used for abdominal organs and kidneys.

We carried out the main anthropometrical, laboratory, and instrumental examinations three times: at baseline, 30 days, and 90 days.

Criteria for the diagnosis. Diagnosis of T2DM according to the criteria of the World Health Organization and International Diabetes Federation (WHO/IDF consultation in 2006) [42]: HbA1c ≥ 6.5% (47.5 mmol/mol), fasting plasma glucose ≥ 7.0 mmol/L, or a patient receives antidiabetic therapy (American Diabetes Association) [30]. Hypertension: systolic BP ≥ 130 and/or diastolic BP ≥ 90 mmHg following repeated examination, or if a patient receives antihypertensive drugs.

### 2.4. Statistics

Justification of the sample size. The estimated treatment difference between comparison groups was set to 10% with a standard deviation of 8% and the superiority margin of 2.5% (δ = 0.025) based on two-sided hypothesis testing. Using SPSS, Sample-Power, V23.0, the number of evaluable individuals required for each treatment arm was ≥20. At least 130 patients were screened and recruited (including nine patients excluded due to statistical outliers), and 95 patients were assessed for eligibility in the comparative clinical trial (Figure 1). The two-sided Student’s *t* test with Bonferroni correction (*p*-value/2) was used, where *p*-values of <0.025 were set as significant differences in intra-groups and <0.025 between groups to compensate for the small number of patients in the groups. The study used SPSS Statistics v23 (SPSS Inc., Chicago, IL, USA) and Microsoft Excel-2023, with normality assessed using histograms and box plots. Given the pilot nature of the study, firm hypotheses were used. The study data are presented in Tables as Mean ± Standard Error of the Mean (M ± SEM) for normally distributed data. All analyses were intention-to-treat.

Correlation–regression analysis was used to find statistical relationships between body weight and HOMA-IR levels before and after the intervention (in percent); it quantifies how changes in one variable correspond to changes in another, indicating the direction and strength of their relationship, including determining the type of correlation, such as positive (variables change in the same direction), negative (variables change in opposite directions), or no correlation (no apparent relationship).

We used electronic databases (Web of Science/Medline, PubMed, Scopus/Science Direct, Google Scholar, EBSCO/Medline Complete, EndNoteClick/Kopernio, and Ovid/Wolter Kluwer, BMJ) for finding research literature.

## 3. Results

Table 1 presents the treatment results of patients in the three comparative groups at baseline and after 90 days of weight loss. Body weight decreased significantly in the Surgery group (−19.8%; *p* < 0.0001) and the VLCD group (−17.4%; *p* < 0.0001), while in the Drug group the decrease was insignificant (−6.5%; *p* = 0.06). The decrease in body weight in all comparison groups occurred due to both fat and lean mass. In Surgery and VLCD groups, the decrease in fat mass from baseline was significant by −29.4% (*p* < 0.0001) and 31.3% (*p* < 0.0001), respectively. Fat-free mass also decreased significantly from baseline in Surgery by −11.7% (*p* = 0.008), but not significantly in VLCD groups by −7.3% (*p* = 0.06).

FBG decreased in the Drug group by −23.2% (*p* = 0.0002), in the Surgery group by −46.5% (*p* < 0.0001), and in the VLCD group by −46.4% (*p* < 0.0001). HbA1c decreased insignificantly in the Drug group by −8.4% (*p* = 0.045), significantly decreased in the Surgery group by −28.4% (*p* < 0.0001), and significantly decreased in the VLCD group by −36.9% (*p* < 0.0001).

HOMA-IR improved significantly in all groups: in the Drug group, decreased by −42.2% (*p* = 0.004); in the Surgery group, by −87.6% (*p* < 0.0001); and in the VLCD group, by −88.7% (*p* < 0.0001), but in the Drug group, HOMA-IR did not reach the normal level (Table 1).

Lipids improved significantly in the Drug group (−8.7% for cholesterol, *p* = 0.004; −17.3% for triglycerides, *p* = 0.005; +12.2% for HDL, *p* = 0.036), in the Surgery group (−17.4% for cholesterol, *p* < 0.0001; −37.7% for triglycerides, *p* < 0.0001; +21.6% for HDL, *p* = 0.002), and in the VLCD group (−23.3% for cholesterol, *p* < 0.0001; −64.5% for triglyceride, *p* < 0.0001; +55.6% for HDL, *p* < 0.0001).

Blood hemoglobin in the Drug group changed insignificantly (+0.5%, *p* = 0.42); in the Surgery group, it significantly decreased (−12.7%, *p* < 0.0001); but in the VLCD group, blood hemoglobin significantly increased (+10.7%, *p* < 0.0001).

SBP in the Drug group significantly decreased by −9.5% (*p* = 0.0002), but DBP decreased insignificantly by −4.1% (*p* = 0.09). In the Surgery group, SBP/DBP significantly decreased by −13.6% (*p* < 0.0001) and by −10.6% (*p* = 0.001), respectively. SBP/DBP in the VLCD group significantly decreased by −23.3% and 21.3%, respectively (*p* < 0.0001).

**Figure 2:**
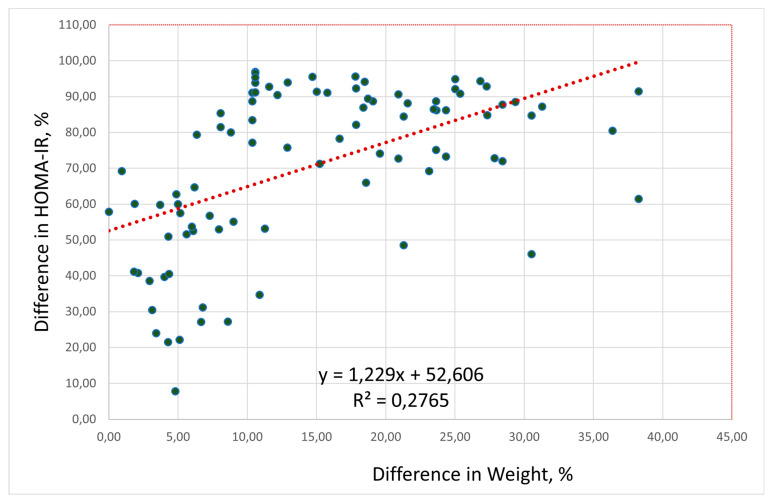
Correlation of differences in % between changes in HOMA-IR and body weight after 90 days of weight loss interventions (r = 0.526; F = 33.2, *p* < 0.0001). Abbreviations: HOMA-IR, Homeostasis Model Assessment insulin resistance index.

In all three compared groups, HOMA-IR decreased during weight loss. We conducted a regression analysis to identify a statistical relationship between weight loss changes and HOMA-IR. Regression analysis of the differences in percent between changes in body weight and HOMA-IR before and after interventions revealed a strong direct positive correlation between weight loss and decreasing HOMA-IR (r = 0.526; F = 33.2, *p* < 0.0001): the greater the weight loss, the lower the HOMA-IR (Figure 2).

According to the data, HOMA-IR positively correlates with weight loss. As a result of regression analysis, the following association was revealed: if body mass decreases by 10%, then HOMA-IR decreases by 65%; if body mass decreases by 25%, then HOMA-IR decreases by 82.6%.

Patients in groups with significant weight loss (−19.8% from baseline in Surgery and −17.4% in VLCD groups) had antidiabetic and antihypertensive medications gradually reduced and discontinued during the first month after baseline due to improvements in metabolic and cardiovascular health. This effect lasted for the remaining two months without laboratory and clinical signs of T2DM and hypertension. Patients in the Drug group were unable to reduce the dosage of their previously taken medications within 90 days.

Side effects in the VLCD group were dizziness, weakness, fasciculation in the lower extremities and abdominal area, and psychological discomfort in the first 2–5 days of weight loss; on 3–10 days of the weight loss, the urine became turbid and dark (urine microscopy revealed the presence of organic salts, such as oxalates/urates/phosphates/carbonates of calcium/magnesium); an increase in body temperature to 38 °C was observed in about half of the patients; sputum expectoration increased 2–3 times more than usual; and looseness of stools and diarrhea.

## 4. Discussion

The study presented positive results of three weight loss methods during 90 days to identify the comparative effects of pharmacologic (semaglutide + empagliflozin), bariatric surgery (endoscopic mini-gastric bypass), and VLCD (analimentary detoxification) on glycemic and other metabolic parameters in patients with T2DM and hypertension. The weight loss interventions resulted in significant reductions in all glycemic parameters (FBG, HBA1c, and insulin) and improvements in lipid (cholesterol, triglycerides, HDL) and blood pressure (SBP and DBP) parameters.

The Surgery and VLCD groups showed a significant reduction in body fat mass compared to baseline. However, they also showed a decrease in lean body mass compared to baseline. In the Drug and VLCD groups, fat-free mass decreased insignificantly. Muscle loss during weight loss is often due to catabolic processes; for example, this was clearly noticeable in the Surgery group, as they also experienced blood hemoglobin loss. This group often has contraindications to effective exercise after surgery [43].

When a person loses weight, they typically lose both fat mass (around 60–80% of the total loss) and fat-free mass (muscle, water, bone mass) (around 20–40%) [44]. A decrease in fat mass is generally accompanied by a decrease in lean mass, and a contributing factor is that the body no longer requires the extra muscle mass to support the lost excess weight. A fatty body requires more muscle and energy for daily activities. The body needs protein to maintain muscle mass and other organs during starvation. The body can use sagging skin as a protein and fuel source during prolonged fasting through a process called autophagy [45].

In the VLCD group, blood hemoglobin increased, whilst they also lost lean mass. Some other studies confirm that weight loss by intermittent fasting results in an increase in hemoglobin and bone mineral density [46]. Blood hemoglobin changed differently in the groups: in the Drug group, it did not change significantly; in the Surgery group, it significantly decreased; and in the VLCD group, it significantly increased. The decrease in blood hemoglobin (anemia) is a common and often delayed consequence after reconstructive bariatric surgery, which alters the anatomical structures responsible for iron metabolism in the body [47].

Calorie restriction can degrade endogenous protein-conjugated substrates in different compartments in the body [48]. Some studies showed that autophagic proteolysis with lysosomal degradation significantly improved the liver and kidney functions and increased blood hemoglobin [49,50,51]. Nutrient deprivation or dietary restriction confers protection against aging and stress in many animals, and induced lysosomal autophagy is part of this mechanism.

The IR parameter improved significantly in all compared groups because it depended on weight loss. Regression analysis showed that the greater the weight loss, the lower the HOMA-IR: if body weight decreased by 10%, then HOMA-IR decreased by 65%; if body mass decreased by 25%, then HOMA-IR decreased by 83%.

The more weight was lost, the better the glycemic data and blood pressure were reduced, and lipids were also improved (cholesterol and triglyceride were reduced, and HDL was increased). Significant improvements in these parameters were clearly observed in Surgery and VLCD groups, i.e., the greater the weight loss, the better these parameters were. Although the Surgery group had the most significant weight loss (−19.8%), anabolic parameters (HDL and blood hemoglobin) were clearly impaired compared to other groups, especially in the VLCD group. For example, HDL and blood hemoglobin levels increased in the VLCD group.

The weight loss interventions had antihyperglycemic, antihypertensive, and lipid-lowering effects, and their effectiveness depended on the level of weight loss.

When insulin-dependent cells deposit excess lipids, their volume increases due to functional hypertrophy (cell enlargement) [52]. Enlarged cells lead to external compression of blood vessels and circulatory disorders [53]. An increase in the cell radius correspondingly increases the path of delivery of oxygen and nutrients from the membrane surface to the center of the cell. To prevent the creation of conditions for the emergence of necrotic processes in the center of the cell (the part furthest from the membrane surface), the transport systems of the cytoplasm will work at the limit of their capabilities, providing this hard-to-reach area with the necessary metabolites [53,54].

The physiological reserves of enlarged cells are reduced, so over time, the entire cell begins to experience a deficiency in synthetic, regulatory, and excretory functions. Ultimately, enlarged adipocytes (hypertrophic cells) lose their functional adaptive value and cease to be useful to the body [2].

Overweight leads to depletion of the pancreatic islet system because the β-cells cannot secrete insulin sufficiently to compensate for the excess weight, and diabetes symptoms worsen [55]. A relative insulin deficiency occurs. After excess weight is lost, the cells’ response to insulin returns to normal [56]. The HI observed in obesity is a secondary phenomenon. Weight loss leads to subsequent normalization of blood glucose and insulin levels in patients with T2DM [24,28].

Insulin synthesis in obesity usually increases proportionally to body weight [57]. Gradually, the body’s compensatory resources are depleted, and its additional synthetic and elimination functions, designed to regulate metabolism in excess biological tissues, are reduced [58].

**Figure 3:**
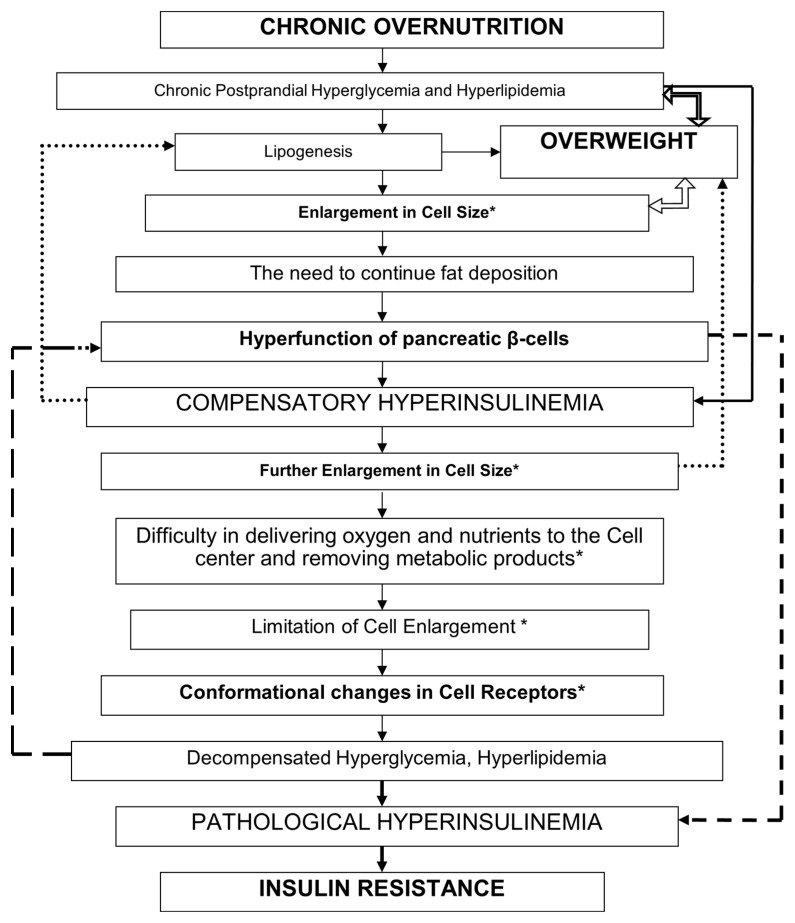
The Concept of insulin resistance development in the context of overweight in type 2 diabetes mellitus. * Adipocytes and other cells of the body with lipid deposition.

HI is a physiological response to every food consumption. Chronic overnutrition and persistent postprandial hyperglycemia and hyperlipidemia lead to overweight (Figure 3). Forced fat accumulation in the body leads to an increase in cell size [52]. Hyperfunction of the pancreatic β-cells leads to compensatory HI. HI occurs as compensation for the forced accumulation of fat by cells. When cells have accumulated fat to the limit, their further increase can threaten their own destruction (death, apoptosis) [2,59]. To limit further nutrient accumulation, HI triggers conformational changes in cell membrane receptors, specifically IR [60]. HI gradually loses its compensatory and adaptive value.

IR is the body’s pathophysiological response to the continued flow of nutrients into the blood (hyperglycemia, hyperlipidemia). IR is a rational response of the body that limits further supply of nutrients to cells. Fat reserves stored in the body themselves require metabolic attention from the body, including adequate blood circulation, thermoregulation, anabolic and catabolic metabolic processes, etc. [60]. IR is a protective mechanism of cells against unsafe fat deposition and excessive energy expenditure [61]. This adaptive-compensatory mechanism is limited by cell size [62]. Under IR conditions, the HI phenomenon suppresses lipolysis, which aggravates the progression of obesity and worsens IR itself [63]. Long-term HI under overeating and overweight depletes the secretory apparatus of pancreatic β-cells, which impairs cellular glucose tolerance. IR prevents further accumulation of fat in cells [64]. A vicious circle arises, in which it is sometimes difficult to understand what is primary and what is secondary. Hyperglycemia, HI, and IR are different links in the same pathogenetic chain of a pathological process.

**Figure 4:**
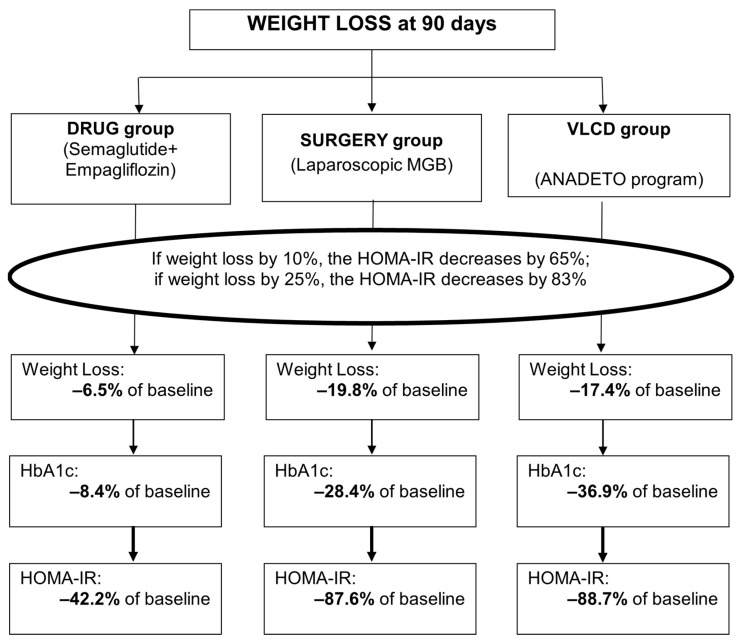
The relationship between weight loss and insulin resistance across Drug, Surgery, and VLCD interventions in patients with type 2 diabetes mellitus and hypertension. Abbreviations: HbA1c, glycated hemoglobin; HOMA-IR, the Homeostasis Model Assessment of Insulin Resistance Index; MGB, minigastric bypass; VLCD, very-low-calorie diet.

Figure 4 shows the relationship between weight loss and IR across three treatment options (the drug, surgery, and VLCD groups) in patients with T2DM and hypertension.

Some studies showed that a moderate decrease in body weight of 5% improved glycemic control, but more substantial weight loss (10–25%) can achieve remission of T2DM [65,66,67]. There are very few studies that directly indicate the level of weight loss at which IR and HI can be completely remitted. Leng et al. (2025) found that progressive 12-month weight loss brought persistent improvements in HI and IR [27].

The body needs to maintain the metabolism of an increased amount of metabolites, which is achieved by increasing the rate of physicochemical processes, for instance, by activating physical (increased blood pressure, body temperature) and/or chemical (increased amount of biologically active substances, hormones, oxidation-reduction reactions) processes that provide cells with increased metabolic intensity [2,68]. Dysfunction of overweight is a prerequisite for the development of IR. Overweight depletes and limits the reserve capacity of the body’s organs and tissues, including pancreatic β-cells. IR, in the context of overweight dysfunction, might be an adaptive cellular response aimed at protecting cells from destruction. IR occurs when cellular spatial reserves are depleted and serves as a defense against excess fat deposition. IR is not a primary but a secondary pathophysiological element in T2DM. We should cope with not the consequence (IR) but the cause—overweight.

## 5. Conclusions

In summary, the greater the weight loss, the greater the improvements in HI and IR across all three treatments (drug, surgery, and VLCD groups) in patients with T2DM and hypertension at 90 days. HOMA-IR was associated with weight loss in patients in the compared groups: HOMA-IR decreases by 65% if body weight decreases by 10%; if body mass decreases by 25%, then HOMA-IR decreases by 83%. Weight loss leads to a reduction in the need for antidiabetic and antihypertensive drugs in patients.

Strengths and limitations: A strength of our study is that it demonstrates for the first time that different weight loss methods may have different effects on HOMA-IR reduction. Published studies comparing the effects of different weight loss interventions (pharmacologic, bariatric surgery, VLCD) on glycemic, lipid, and blood pressure parameters in people with T2DM and hypertension are very limited in scope and number. We attempted to make a research contribution to the study of the role of insulin resistance in the development of T2DM.

This study has several limitations. First, the study included a relatively small number of patients with T2DM and hypertension. Second, the clinical study had approximately 14% of the randomly assigned population drop out prior to completion. There was only a 90-day study; that is not enough to observe T2DM and hypertension outcomes. Third, the protective role of insulin resistance is a new understanding of the pathophysiology of the development of T2DM in the context of overweight, and the results focus more on the overall effect across the three groups than on a direct comparison between them.

Further high-quality multicenter clinical trials with a large sample size and longer-term follow-up are needed to confirm and extend the results of the study.

## Figures and Tables

**Table 1:** Comparative changes in body weight, glycemia, insulin, HOMA-IR, HbA1c, lipids, and blood pressure in Drug, Surgery, and VLCD clinical groups at baseline and 90 days after weight loss treatment (*n* = 90) (M ± SEM).

Variables	Drug Group (*n* = 30)		Surgery Group (*n* = 30)		VLCD Group (*n* = 30)	
	Baseline	90 Days	Baseline	90 Days	Baseline	90 Days
Age, years	48.1 ± 1.5	-	45.6 ± 1.9	-	49.5 ± 1.7	-
Body weight, kg (% of Baseline)	105.7 ± 3.1	98.8 ± 3.0 (−6.5%)	112.4 ± 3.3	90.2 ± 2.9 *** (−19.8%)	109.3 ± 3.2	90.3 ± 2.9 *** (−17.4%)
Fat mass, kg	43.2 ± 1.9	38.7 ± 1.9	46.3 ± 1.9	32.7 ± 1.8 ***	46.4 ± 1.9	31.9 ± 1.8 ***
Fat-free mass, kg	62.4 ± 2.1	60.1 ± 2.1	66.1 ± 2.1	58.4 ± 2.1 **	62.9 ± 2.1	58.3 ± 2.0
HbA1c, % (% of Baseline)	9.12 ± 0.31	8.35 ± 0.31 (−8.4%)	9.41 ± 0.34	6.74 ± 0.29 *** (−28.4%)	9.94 ± 0.34	6.27 ± 0.28 *** (−36.9%)
Fasting blood glucose, mmol/L	9.14 ± 0.38	7.02 ± 0.35 **	9.98 ± 0.45	5.34 ± 0.33 ***	10.11 ± 0.46	5.42 ± 0.31 ***
Insulin, nU/L	28.6 ± 2.2	21.6 ± 2.0 *	26.7 ± 2.1	6.27 ± 0.35 ***	28.03 ± 2.2	6.11 ± 0.36 ***
HOMA-IR (% of Baseline)	11.6 ± 1.3	6.7 ± 1.1 ** (−42.2%)	12.2 ± 1.34	1.51 ± 0.09 *** (−87.6%)	12.9 ± 1.36	1.46 ± 0.08 *** (−88.7%)
Cholesterol, mmol/L	5.85 ± 0.12	5.34 ± 0.13 **	5.95 ± 0.11	4.93 ± 0.11 ***	6.09 ± 0.11	4.67 ± 0.08 ***
Triglycerides, mmol/L	2.02 ± 0.09	1.67 ± 0.09 **	2.28 ± 0.08	1.42 ± 0.07 ***	2.37 ± 0.09	0.84 ± 0.08 ***
HDL, mmol/L	0.98 ± 0.04	1.1 ± 0.05	0.97 ± 0.04	1.18 ± 0.05 **	0.88 ± 0.04	1.37 ± 0.06 **
Hemoglobin, g/L	135.2 ± 2.3	135.8 ± 2.2	138.4 ± 2.1	120.7 ± 2.0 ***	127.6 ± 2.1	141.2 ± 1.9 ***
SBP, mmHg	144.5 ± 2.3	130.8 ± 2.3 **	148.7 ± 2.8	128.4 ± 2.2 ***	158.6 ± 2.7	121.7 ± 2.2 ***
DBP, mmHg	92.7 ± 1.9	88.9 ± 2.1	97.4 ± 2.1	87.6 ± 1.9 **	101.4 ± 1.97	79.8 ± 1.3 ***

* *p*-values < 0.025, ** *p* < 0.01, and *** *p* < 0.0001 were significant compared with baseline (before treatment) in all groups. Abbreviations: HbA1c, glycated hemoglobin; HDL, high-density lipoprotein; HOMA-IR, the Homeostasis Model Assessment of Insulin Resistance Index; M, mean; SBP/DBP, systolic/diastolic blood pressure; SEM, standard error of the mean; VLCD, very-low-calorie diet.

## Data Availability

The data are available from the authors upon reasonable request. Those wishing to request the study data should contact the Principal Investigator of a research grant: Dr. Oshakbayev Kuat (Emails: okp.kuat@gmail.com; kuat.oshakbayev@umc.org.kz, phone: +7 7013 99-93-94).

## Supplementary Materials

The following supporting information can be downloaded at https://www.mdpi.com/article/10.3390/jcm15020546/s1, File S1: PROTOCOL trial_T2D_CVD_Eng; File S2: CONSORT Extension_Treatment Checklist.

## Abbreviations

ANADETO	analimentary detoxication
BMI	body mass index
FBG	fasting blood glucose
GLP-1RA	glucagon-like peptide-1 receptor agonists
HbA1c	glycosylated hemoglobin
HDL or LDL	high- or low-density lipoprotein
HI	hyperinsulinemia
HOMA-IR	Homeostasis Model Assessment of Insulin Resistance Index
IR	insulin resistance
MGB	minigastric bypass
SBP or DBP	systolic or diastolic blood pressure
SGLT-2i	sodium-glucose cotransporter-2 inhibitor
T1DM or T2DM	type one or two diabetes mellitus
VLCD	very-low-calorie diet
