# Supplementary material for: Changes in Insulin Resistance with Different Weight Loss Methods in Patients with Type Two Diabetes Mellitus and Hypertension: A Comparative Clinical Trial
Source: J Clin Med. 2026 Jan 9;15(2):546. doi: 10.3390/jcm15020546 (PMC12842082; doi:10.3390/jcm15020546)
Supplement: Supplementary file 1 [file jcm-15-00546-s001.zip › Supplementaries/1_PROTOCOL trial_T2D_CVD_Eng.docx]

**SCIENCE COMMITTEE OF THE MINISTRY OF SCIENCE AND HIGHER EDUCATION OF THE REPUBLIC OF KAZAKHSTAN**

**CORPORATE FUND «UNIVERSITY MEDICAL CENTER»**

Decision of the National Scientific Council: protocol #112 from 30 July, 2024

Agreement # 315-ГФ24-26 of 09.09.2024

**PROTOCOL**

**of scientific research grant for 2024-2026 # AP 23488544 on the title "Study of the effectiveness of pharmacological, surgical and dietary methods of weight loss on the life expectancy of patients with metabolic syndrome:**

**a comparative clinical study"**

Priority: Life and Health Science.

Sub-priority: Reducing the burden of socially significant diseases.

Total project cost: 119 927 351 tenge, including by year:

- for 2024 - in the amount of 38,435,203 tenge;

- for 2025 - in the amount of 40 224 600 tenge;

- for 2026 - in the amount of 41 267 548 tenge.

PI: Oshakbaev Kuat, MD, professor, therapist of the highest category

**Astana, 2024**

1. **Brief overview of the protocol**

| Title of the study | Effectiveness of pharmacologic, surgical, and dietetic weight loss methods on lifespan in patients with metabolic syndrome: a comparative clinical trial. |
| --- | --- |
| Grantor | Science Committee of the Ministry of Science and Higher Education of the Republic of Kazakhstan |
| Duration and Location of the Study | For 2024-2026.  Clinical centers of the University Medical Center (UMC).  Astana Medical University.  Genomic studies in the laboratory of Nazarbayev University.  Patient recruitment at the clinical sites of the UMC and outpatient clinics in Astana. |
| Purpose | Reducing individual excess body fat leads to increased life expectancy |
| Aim:  Project objectives | To study lifespan, glycemic and lipid metabolism, quality of life, and cost-effectiveness of pharmacologic, surgical, and dietetic weight loss methods in obese patients with MS at 24 weeks in a comparative clinical trial.   1. To compare glycemic and lipid metabolism in the patients after a pharmacological, surgical, and very-low-calorie diet (hereafter VLCD). 2. To determine leukocyte telomere length in the patients after pharmacological, surgical, and VLCD. 3. To study clinical side effects in the patients after pharmacological, surgical, and VLCD. 4. To conduct quality of life and cost-effectiveness analysis in the patients before/after pharmacological, surgical, and VLCD.   . |
| Study design | A 24-week open label, prospective, multicenter, comparative clinical trial with the intention-to-treat analysis. |
| Participants | Totally 150 adult patients with MS distributed in three comparative groups: drug treatment, surgery and VLCD (each 50 patients). |
| Inclusion criteria | 1) written informed consent;  2) T2D≥3-year with glucose lowering therapy including insulin;  3) 30-60 years old;  4) BMI≥27 kg/m2 for both sex, for Asian ethnicity;  5) ongoing treatment with antihypertensive treatment;  6) weight loss 15-20% at baseline and dynamic follow-up up to 24 weeks. |
| Exclusion criteria | 1) T1DM;  2) <30 age >61 years old;  3) patients after bariatric surgery;  4) unstable cardiac disorders (New York Heart Association class IV heart failure, refractory angina, uncontrolled arrhythmias, critical valvular heart disease, or severe uncontrolled hypertension);  5) glomerular filtration rate <40 mL/min and/or dialysis within 14 days before screening;  6) ejection fraction <40%;  7) history of alcohol consumption >30 g/day within the past 3 years;  8) malignancy within the past 5 years;  9) gestation or lactation;  10) hereditary diseases;  11) known hypersensitivity to any of the test substances. |
| Interventions | 1. The 1st group (n=50) receives subcutaneous Semaglutide (GLP-1RA) 1 mg once a 7 day with oral Empagliflozin (SGLT-2i) 25 mg once a day that is a novel combination additionally to standard medical treatment including anti-diabetes, antihypertensive, lipid-lowering, symptomatic therapy. 2. The 2nd group (n=50) receives surgical method (laparoscopic MGB). These patients pass through additional pre-operation examination (blood tests and electrocardiography, esophagogastroscopy, ultrasound, and other necessary standard methods). 3. The 3rd group (n=50) receives very-low-calorie-restriction diet (‘Analimentary-detoxication’) including <100 kcal/day with fat-free vegetables (tomato/cucumber) and salt intake (5-6 g/day), optimum physical activity, and sexual self-restraint. [1-3] The program goals the next outputs: a) use of own fatty store; b) control endogen intoxication; c) reuse of interim metabolic substrates. |
| Key performance parameters: | *Primary endpoints*:  weight loss 15-20% from baseline;  leukocyte TL.  *Secondary endpoints*:  HbA1c;  fasting blood glucose and 2-hour oral glucose tolerance test (OGTT); systolic/diastolic BP;  lipid profile;  immunoassay insulin in blood;  HOMA-IR,  adverse events. |
| Basic security parameters | Strict adherence to inclusion and exclusion criteria. |
| Consent to treatment | Before treatment, the physician must explain the following to the patient:  1. The purpose, methods, and duration of treatment.  2. Predicted benefits and side effects.  3. Alternative treatments.  4. The use of comparable treatments does not harm the patient's interests.  5. The patient has the right to choose or refuse treatment.  6. After the patient's written consent for inclusion in the scientific study and the intervention has been obtained, treatment can begin. |
| Side effects | Adverse event analysis: symptoms and signs, dates of detection and resolution, severity, nature of the procedure, and relationship to the test methods are recorded.  Relationship judgments are categorized as: definite, possible, impossible, no relationship, and uncertain relationship. |

1. **Research team:**

| **№**  **п/п** | **Full name (if any), education, degree, academic rank** | **Main place of work, position** | **Hirsch index, Researcher ID, ORCID, Scopus Author ID (if available)** | **Role in the project or program and the nature of the work to be performed** | **Brief justification for participation** |
| --- | --- | --- | --- | --- | --- |
|  | Оshakbayev Kuat Pernekulovich, MD, PhD, DMSc., full-professor in Medicine, General Practitioner of the highest degree | CF “University Medical Center”, Department of Internal Medicine,  Deputy director of science & education | H-index-5 by WebOfScience (Clarivate).  ResearcherID: D-1959-2011.  ScopusAuthorID: 57200682883  ORCID:0000-0003-4883-295X | PI. Research manager. Conducting VLCD clinical study. | The general research manager and coordinator of the scientific and practical activities. Conducting a clinical trial (non-drug treatment). Writing articles and patent for invention. |
|  | Bikhanov Nurzhan Aktleuovich, MD, PhD, Surgeon of the highest degree | UMC, Department of Surgery, Head of multidisciplinary surgery program | ORCID.org/0000-0002-0890-4763 | Main researcher | Conducting a clinical trial (surgical treatment), coordinator. Scientific analysis. Writing articles and an invention patent. |
|  | Idrissov (Idrisov) Alisher Saugabayevich., MD, PhD, D.M.Sc., Endocrinologist of the highest degree | Astana Medical University, Department of Internal Medicine #2, Associate Professor | H-index-2 by WebOfScience (Clarivate).  ScopusID: 56974370300.  ORCID.org/0000-0003-2006-7803 | Main researcher | Conducting a clinical trial (drug treatment), coordinator. Scientific analysis. Writing articles and an invention patent. |
|  | Issabayeva Assel Erbolovna.  MD, Endocrinologist. Bachelor in medicine | UMC, Department of Internal Medicine,  Resident Doctor of the 2^nd^ year, | ORCID: 0000-0003-1082-3687 | Researcher | - Recruitment and management of patients at all stages of outpatient and inpatient follow-up;  - records management;  - participation in scientific publications |
|  | Slyamova Gulnur Nurlanovna,  MD, Endocrinologist. Bachelor in medicine Master of Public health | UMC, Department of Internal Medicine,  Resident Doctor of the 1^st^ year | - | Researcher | - Recruitment and management of patients at all stages of outpatient and inpatient follow-up;  - records management;  - participation in scientific publications |
|  | Suleimenov Timur Sakenovich.  MD, Surgeon | UMC, Department of Surgery, Multidisciplinary surgery program, Surgeon.  Astana Medical University,  department assistant. | ORCID ID: [0000-0003-1672-6722](https://orcid.org/0000-0003-1672-6722) | Researcher | - Recruitment and management of patients at all stages of outpatient and inpatient follow-up;  - records management;  - participation in scientific publications |
|  | Gusmanov Arnur, MD, MPH, a senior biostatistics | Nazarbayev University. Nazarbayev University School of Medicine. Instructor of Department of Medicine | H-index-4 (WebOfScience).  ResearcherID: AAV-3524-2021.  ScopusAuthorID: 57216565668  ORCID: 0000-0003-2805-8645. | Leading Researcher | Biostatistician, methodologist (Machine learning pipeline and modelling. Deep learning pipeline). Review of written articles. |
|  | Nabiyev Altay Nugmanovich, MD, Ph.D., General practitioner of the highest degree | UMC, Internal Medicine, Department of Internal Medicine,  Director | ORCID: 0000-0002-0925-3528 | Leading Researcher | Physician-coordinator of clinical trials (drug, surgical and non-drug treatment). |
|  | Daniyarova Gulnur Daniyarkyzy, MD, cardiologist | UMC, Department of Science, General manager | - | Manager | Leading physician-coordinator of population and clinical studies. |
|  | PhD student | UMC | - | PhD student | - Recruitment and management of patients at all stages of outpatient and inpatient follow-up;  - records management;  - participation in scientific publications |
|  | PhD student | UMC | - | PhD student | - Recruitment and management of patients at all stages of outpatient and inpatient follow-up;  - records management;  - participation in scientific publications |
|  | PhD student | UMC | - | Accountant, economist | Financial and economic support |

1. **Background**

The prevalence of T2D in the world is constantly increasing. T2D increases the risk of cardiovascular complications by 5-10 times especially in older age groups. Obesity is a major and multidimensional health problem worldwide and a major risk factor for additional health concerns such as cardiovascular diseases, T2D, metabolic syndrome (MS) and some cancers. Objective modest weight loss (5%-10%) is clinically meaningful in patients with MS.

Obesity is a major and multidimensional health problem worldwide and a major risk factor for additional health concerns such as cardiovascular diseases, T2D, microalbuminuria, fatty liver disease, inflammatory diseases, as well as many types of cancer, creating a continuum of MS, reducing life expectancy. [4, 5]

Over the past 20 years, the prevalence of T2D in the world has been constantly growing. [6] High incidence of T2D is associated with a pandemic of overweight. [7] Patients with T2D have a high risk of cardiovascular complications by 5-10 times, [8, 9] and it is the main cause of death. [10, 11] Obesity accelerates aging. [5]

Although lifestyle changes are seen as a crucial part of MS treatment, supplementary pharmaceutical/surgery interventions are often needed. As obesity is a chronic disease, drug products or surgуical procedures need to be both efficacious and safe for long term use. [12]

Approximately 60% of adults in the US have obesity. [13] In Kazakhstan every fifth of adult men (21.4%) is now obese. [www.globalnutritionreport.org] Obesity increases the incidence and mortality from chronic diseases. [14, 15] Despite these knowledge, definitive data on the effect of intentional weight loss through interventional studies and the chronic diseases risk reduction are lacking. Bariatric surgery could be the effective currently available treatment for obesity despite his side-effects. [16, 17]

Nowadays, we all know that weight loss is one of the main purposes to improve health and decrease the clinical outcomes. [17] It's known there several types of weight loss as pharmacologic, surgical, and diets. Although almost every method of weight loss has a benefit for health independently, it is still debated which of these methods provides the greatest benefit on clinical and lab outcomes, lifespan, side effects, quality of life, and the cost-effectiveness of those interventions are being still discussed. The economic burden for the health care system is determined by the high cost of medical interventions aimed at correcting the various clinical components of metabolic syndrome, as well as by the significant indirect costs associated with productivity losses which leads to public health authorities searching for new strategies for treating obese patients with T2D and AH.

Objective modest weight loss (5%-10%) is clinically meaningful in patients with MS. However, greater weight loss may be required to achieve improvements in or remission of certain weight-related complications. [18] This study devoted to reach the effect of large weight loss (- 15-20% from baseline). Most studies reporting large weight loss and relevant outcomes used bariatric surgery or lifestyle modifications. [19, 20] Improvements in glucose metabolism and cardiovascular risk factors were observed in patients who achieved large weight loss through lifestyle interventions or pharmacotherapy. [17, 21] Bariatric surgery compared with no surgery was associated with a significantly lower incidence of obesity-associated cancer and cancer-related mortality. [16] Large weight loss should be the main treatment target on obesity-related complications and for patients with severe obesity.

Weight loss methods decrease in multiple cardiovascular risks, reduce in medication, improve metabolism. [22, 23] Weight loss in patients leads to cost savings by reducing the number of doctor visits, prescription drug tests, sick days, emergency visits and hospitalizations, but also has more long-term economic benefits, and reducing a risk for a wide range of chronic diseases. [24]

Telomeres are DNA structures at the end of chromosomes that protect them from damage and instability. Telomeres shorten with each cell cycle; thus, telomere shortening indicates the proliferative history of the cell. [25, 26] When telomeres become critically short, cells enter senescence cell cycle arrest or undergo apoptosis. Age, sex and genetics are associated with telomere attrition, but lifestyle factors have also been associated with telomere shortening. [27, 28]

Lifestyle factors such as smoking, lack of physical activity, obesity, stress, exposure to pollution, etc. can potentially increase the rate of telomere shortening, cancer risk, and pace of aging. Dietary restriction, appropriate diet (high fiber, plenty of antioxidants, lean/low protein, adding soy protein to diet), and regular exercise can potentially reduce the rate of telomere shortening, disease risk, and pace of aging. [25, 26] Authors found a strong correlation between the telomere shortening rate and the life span. [29]

TL is considered to have a critical role in the cell regulation and cell death. TL can be a reliable indicator of biological aging and of the risk of developing age-related chronic diseases. Telomere shortening has been associated with a broad range of pathologies, including lung, liver, hematologic, T2D, cardiovascular diseases, and a lot of types of cancer [30, 31]. Leukocyte TL is considered as a prognostic marker for cardiovascular diseases and T2D. [32] Telomere shortening was found in overweight children, which also has an effect on biological age. [27] An inverse correlation between TL and insulin resistance and BMI was established. [28] Assessment of change in TL may be a useful marker of the effectiveness of weight loss methods in a lot of non-communicable chronic diseases. *There is a great range in normal TL in different people, but within the same person TL naturally shortens with disease and with age.*

The effective scale-up treatment of obese patients with MS is expected to increase their lifespan, improve the results of conventional treatment, innovate the health care system and the society at large. The benefits of treatment methods of morbid obesity are expected to overcome their expenses, becoming cost-effectiveness, however, there is no evidence on which of those interventions may be more efficient to implement.

Currently, we have accumulated positive results in the weight loss treatment of obese patients with T2D and AH methods by pharmacologic [33, 34], very-low-calorie diet (VLCD) as ANADETO, [2, 35, 36] and surgical (Vertical Sleeve Gastrectomy, or Minigastric bypass, etc.) weight loss [37, 38] more than 10% from baseline weight. However, study of lifespan, and side effects, glycemic/lipid metabolism, quality of life, and cost-effectiveness of patients with MS after pharmacologic, surgical, and dietetic weight loss methods in a comparative clinical trial has not been conducted worldwide.

The main idea of ​​the study project is to show the comparative results of different weight loss methods as such pharmacological, surgical, and dietic, in reduction in body weight 15-20% from baseline.

1. **Research methods and ethical issues**

**Study design:** A 24-week open label, prospective, multicenter, comparative clinical trial with the intention-to-treat analysis.

The appropriate national and institutional regulatory authorities and ethics committees will approve the study design, and all participants will provide written informed consent. The study will fulfilled in University Medical Center together with Nazarbayev University School of Medicine and National Laboratory Astana and Astana Medical University.

**Participants.** Totally 150 adult patients with MS distributed in three comparative groups: drug treatment, surgery and diet therapy.

The study design is prospective because we have to comparatively measure TL before/after interventions; the study may not be an observation study. Because of the trial will non-randomized patients will be allocated to each group based on equal baseline characteristics according to the inclusion/exclusion criteria and including adjustments for baseline and confounding characteristics.

The patients will be allocated to each group based on the patient and two physicians (surgeon and therapist) decisions to avoid the risk of "selection bias". Normalization of parameters will focuse on the work required to embed processes into statistics.

Despite the many advantages of randomized trial design, there are several misconceptions particularly in interdisciplinary comparing very different treatment methods as surgical, drug, non-drug. These misconceptions include: heterogeneity of study interventions, the length of treatment, different compared methods, appraisal and critique, pharmaceutical industry influence, ethical standards, etc. [39, 40]

Randomization and blinding are not possible within the study due to the study design. The comparative methods have different interventions, allocation of the patients cannot be ethically performed, informed consent is impossible to blind; results may not always mimic comparative treatment methods; randomization requires clinical equipoise. [41, 42]

***Inclusion criteria*:** 1) written informed consent; 2) T2D≥3-year with glucose lowering therapy including insulin; 3) 35-55 years old; 4) BMI≥27 kg/m2 for both sex, for Asian ethnicity; 5) ongoing treatment with antihypertensive treatment; 6) weight loss 15-20% at baseline and dynamic follow-up up to 24 weeks.

All included patients before recruiting to the study received standard-of-care treatment for T2D and AH according to regional guidelines.

***Exclusion criteria:*** T1D; <34 age >56 years old; unstable cardiac disorders (New York Heart Association class IV heart failure, refractory angina, uncontrolled arrhythmias, critical valvular heart disease, or severe uncontrolled hypertension); glomerular filtration rate <30 mL/min and/or dialysis within 14 days before screening; malignancy within the past 5 years; gestation or lactation; hereditary diseases; known hypersensitivity to any of the test substances.

*The 1^st^ group* (n=50) receives subcutaneous Semaglutide (GLP-1RA) 1 mg once a 7 day with oral Empagliflozin (SGLT-2i) 25 mg once a day that is a novel combination additionally to standard medical treatment including anti-diabetes, antihypertensive, lipid-lowering, symptomatic therapy.

*The 2^nd^ group* (n=50) receives surgical method (laparoscopic MGB). These patients pass through additional pre-operation examination (blood tests and electrocardiography, esophagogastroscopy, ultrasound, and other necessary standard methods).

*The 3^rd^ group* (n=50) receives very-low-calorie-restriction diet (‘Analimentary-detoxication’) including <100 kcal/day with fat-free vegetables (tomato/cucumber) and salt intake (5-6 g/day), optimum physical activity, and sexual self-restraint. [1-3] The program goals the next outputs: a) use of own fatty store; b) control endogen intoxication; c) reuse of interim metabolic substrates.

A combination of in-person conversations and telephone calls conduct during the study period. (Fig. 1, CONSORT-Flow-Diagram).

## Pharmacol-group (n≤50)

## Surgery-group (n≤50)

## VLCD-group (n≤50)

## Follow-up

## Analysis

24 weeks

Included to intention-to treat analysis

## Enrollment

Assessed for eligibility (n≥ 150)

Allocation (n≤150)

Consider of Excluded

- Refused to treat
- Moved to live on another place
- Excluded due to noncompliance

Lost to follow-up with given reasons

Fig. 1. CONSORT Flow Diagram.

**Justification of the sample size*.*** The estimated treatment difference will set to 10% with a standard deviation of 8% and the superiority margin of 5% (δ=0.05) [43] based on two-sided hypothesis testing. Using SPSS,Sample-Power,V23.0, the number of evaluable individuals needed per treatment arm >32. At least 150 patients will be screened and recruited in the comparative clinical trial. [44]

***End Points:*** *The primary endpoints:* reduction in body weight 15-20% from baseline; leukocyte TL. *The secondary endpoints:* HbA1c; fasting blood glucose and 2-hour oral glucose tolerance test (OGTT); systolic/diastolic BP; lipid profile; immunoassay insulin in blood; adverse events.

**Interventions**

*One of the methods* that will be used in project is a new drug combination for weight loss in patients with T2D – Glucagon-like-peptide-1-receptor-agonists with sodium–glucose-co-transporter-2-inhibitors . [34, 45, 46] Guidelines on hyperglycemic management recommend either GLP-1RAs or SGLT-2is as second-line treatments for patients with T2D on metformin monotherapy. The use of both antidiabetic drugs in combination is rare in the literature. [46]  During the last decade, the results of large-scale, randomized, clinical trials on newer antidiabetic agents, GLP-1RA and SGLT-2 inhibitor have been shown to modify cardiovascular risk factors, such as insulin resistance, body weight, BP, and lipid profile, additionally to induce weight loss. [47-49]

*The second method* is a bariatric operation – minigastric bypass (MGB) that is endovideososcopic techniques with the intraperitoneal using of synthetic/biological materials. MGB is performed to reduce the absorption surface of the gastrointestinal tract by shunting the greater part of the stomach, the duodenum and the initial section of the small intestine, which reduces the absorption of food and leads to a decrease in the production of gastrointestinal hormones. [37, 50, 51] Also, along with the normalization of carbohydrate metabolism, the level of lipids in the blood is effectively reduced.

*Third method* for weight loss that will be used in project is a fast weight loss program “Analimentary detoxication” based on VLCD, fat-free vegetables and salt consumption, and optimum physical activity. [2, 52, 53]

Every presented method for weight loss in obese patients with T2D and AH have the pros and cons. The objective of the weight loss methods is to maintain an achieved target weight at 24 weeks, study of biomarkers of aging, glycemic and lipid metabolism, quality of life, and cost-effectiveness, which will allow to develop evidence-based algorithms for management of patients with MS.

In case of successful achievement of the study’s purpose we could show first results of effectiveness of weight loss methods including pharmacologic, surgical, and dietetic in patients with MS in a comparative clinical trial, and to evaluate their effects on lifespan, glycemic and lipid metabolism, side-effects, [54] quality of life, and cost-effectiveness. The results can give recommendations to health policy makers, endocrinologists, cardiologists, bariatric surgeons, dietitians, researchers, and obese patients with T2D with AH to provide the best treatment way and make decision to fight the chronic diseases. Cost-effectiveness assessment will help to support health policy decisions regarding these alternatives.

***Justification of the sample size.***

The estimated treatment difference between comparison groups was set to 10% with a standard deviation of 8% and the superiority margin of 5% (δ=0.05) [43] based on two-sided hypothesis testing. Using SPSS,Sample-Power,V23.0, the number of evaluable individuals needed per treatment arm ≥32. At least 150 patients will be screened and enrolled in the study.[44]

**End Points:** Primary endpoints: reduction in body weight 15-20% from baseline; leukocyte TL.

Secondary endpoints: HbA1c; fasting blood glucose and 2-hour oral glucose tolerance test (OGTT); systolic/diastolic BP; lipid profile; immunoassay insulin in blood; HOMA-IR; adverse events.

**V. METHODS**

- **Criteria** **for the diagnosis of MS.** Diagnosis of MS (IDF-2005). Diagnosis T2D: HbA1c≥6,5%, fasting plasma glucose level ≥7.0 mmol/l, or a patient receives antidiabetic therapy [ADA, 2023]. [55] AH: systolic-BP≥130 and/or their diastolic-BP≥90 mm.Hg following repeated examination, or a patient receives antihypertensive-drugs.
- **General obesity** is measured by determining of BMI (kg/m^2^). Abdominal obesity is assessed by waist circumference using the standards for the Asian nationality by IDF(2018). [56]
- **Body composition parameters** including fat mass, visceral fat, fat-free mass, total body-water, muscle/bone mass, metabolic age, basal-metabolic-rate, and bioimpedance will be evaluated by Tanita-MC-980MA Multi-Frequency Segmental Body-Composition-Analyzerer(TanitaCorp,Japan).
- **Physical activity** will be assessed as the number of steps taken by patients, as determined by individual pedometers from Hoffmann-La-Roche(Switzerland) or other individual digital system.
- **Laboratory study.** On the same blood samples, standard laboratory a complete blood count, erythrocyte sedimentation rate, urea, creatinine, glucose, electrolytes, HbA1c, lipid profile (total-cholesterol, HDL/LDL, triglycerides), total proteins, coagulation profile, bilirubin, hepatic enzyme activities, blood/ urine ketone bodies.
- **Hormones assay.** Fasting serum insulin by immunoassay method (CzechRepublic).

The Homeostasis Model Assessment insulin resistance indexes (HOMA-IR) will be used as a surrogate measure of insulin sensitivity as follows: HOMA-IR=((fasting insulin in nU/L)×(fasting glucose in mmol/l)/22.5).

- **Measurement of TL.** TL will be measured in T/S ratio with an adaptation of the quantitative polymerase chain reaction (q-PCR) methods. The intraassay and interassay variability (CV) for quantitative polymerase chain reaction will be shown. [57]

Blood samples for measurement of TL will be collected in sterile vacutainer and delivered to the Genom and personalized medicine laboratory (Nazarbayev University). DNA was purified using Promega Wisard DNA system in -20 С. In short, for measurement of telomere repeat copies, primers will be added. Two control samples will be run in each experiment to allow for normalization between experiments, and periodical reproducibility experiments will be performed to guarantee correct measurements. The ratio calculated is proportional to average TL per cell.

- **Quality of life assesses with international questionnaire BAROS** Bariatric Analysis and Reporting Outcome System, EuroQoL.
- **Cost-effectiveness analysis** conductes using as outcomes both clinical as well as EuroQol to estimate the incremental cost-effectiveness of the three alternative interventions. Analysis considers specific costs (a) direct medical costs (including the intervention itself and follow-up care to the condition), (b) indirect nonmedical costs, and (c) additional non-medical costs. Costs of medical care obtaines from economic departments of participating medical centers identifying resources consumed during the interventions. Indirect costs obtaines from patients. The limited time horizon of this study will make it necessary to use a model to extrapolate the observed effect of the treatments assessed in the long term.
- **Consultations of specialists** conductes before/during/after the study 24-week period.
- **Imaging methods**: Ultrasound of internal organs (GE Vivid 7 Ultrasound; GE Healthcare Worldwide USA, Michigan); ECG.

**Interpretation of the data.** The primary hypothesis is that patients treated with the comparative methods would experience a greater weight reduction and improving in the primary/secondary endpoints; then the groups selects to compare between themselves in the aggregate using multiple linear regression analysis. To establish the effectiveness of the comparative methods based on the principle: H0 (null hypothesis) one method doesn’t differ from another/other; and Ha (alternative) one method differs from another/other.

**Statistical analysis.** The statistical analysis plan considers non-compliance, loss-of-follow-up, and missing-data. Multiple-logistic-regression-models, ANOVA/ANCOVA uses to predict the probability of specific variables. Mixed-effect-models or generalized-estimating-equations (GEE) evaluates with statisticians. The Student's two-factor-t-test, OR (95%,CI), Pearson's criterion-χ2 will be used to assess differences in proportions. Mann-Whitney U-test and Kruskal-Wallis test are used for nonparametric. Pearson or Spearman correlation coefficient (r). To determine dependent/independent variables and to control confounders discusses between the team using literature data.

Regression analysis performs using state of the art machine learning algorithms in python code. These include application of algorithms such as KNN, SVM and Random Forests. Additionally, we will apply deep learning methodology for creating a model to test the hypothesis testing as a binary classifier. This learning model uses neural network, given new data will be able to make personalized effects of the three treatment methods. Study data presentes in tables as M±SE or median (25-75%) based on distribution of variables. Survival analysis (Kaplan-Meier or Cox-regression) uses to assess mortality in the three comparative treatments. Statistical analysis using SPSS for Windows,ver.28.0.

**Ethical issues.** The project was approved by the local bioethic committee (LEC) to conduct the study (Protocol #7/ПЭ, October-11-2023, project #2023/01-24) which recommended a non-randomized clinical trial design. The study conducts in accordance to Helsinki Declaration and ICH GCP as well as the guidelines for preparation of CONSORT; and will be registered in the US/EU clinical-trial registries (ClinicalTrials.gov or in ЕС ClinicalTrialsRegisters). Each patient will have a voluntary right to participate in the study with signed consent form. GCP standards, patient safety procedures follows in accordance with the requirements of JointCommissionInternational.

Informed consent will be independently administered by three researchers from each study groups and the LEC staff will monitor. Data and database management plan will be approved by the LEC. Data confidentiality will be protected by storing research data on password protected computers in locked cabinets. Limiting access to study data to only a few team’s members (PI and biostatistics). De-identifying data using encrypted systems for storing research data.

After the three-year study, patients will be monitored to review the results for further research.

To protect intellectual property, the following will be created: patents for inventions, articles in journals with an impact factor in the Q1-Q3 (WebOfScience and/or CiteScore/Scopus percentile >50, practical guidance. International principles of copyright law, which do not allow plagiarism/fabrication/falsification.

**Data Storage and Confidentiality.** All data obtained during the study will be kept confidential and stored by the project manager for the duration of the study and for five years afterward. To ensure data confidentiality, data encryption will be performed where necessary, and an appropriate storage mechanism will be selected to prevent unauthorized access. Access to the data will be limited to individuals designated by the project manager.

International copyright and intellectual property requirements will be observed. There will be no copyright infringement, falsification, fabrication, or plagiarism (false authorship). The use of research results, data, and metadata by individual participants without the consent of other team members will not be permitted.

Several methods will be used to protect intellectual property: domestic and foreign patents, intellectual property certificates, publication of articles in international journals with a high impact factor, and practical guidelines. Data obtained during the study will be stored on the personal computers (hereinafter referred to as PCs) of the study director and co-director, and will also be presented in international databases (e.g., SciNote or DMP). Data security is ensured by official registration of the research in international databases (priority determination), as well as limited access to PC data using passwords.

Each member of the VSC will have an individual work schedule in accordance with the technical specifications and the work schedule for the corresponding year of the grant research (the technical specifications and work schedule are attached).

**VI. Research environment**

1) **The applicant of the project is the Corporate Fund "University Medical Center" (UMC)** [https://umc.org.kz/en/]. The UMC will coordinate the project during the entire duration of the project, and will carry out the main part of the clinical recruitment of material through epidemiological and clinical sampling, processing of primary clinical material, and research activities.

UMC will provide clinical, scientific and laboratory facilities for the implementation of the Project. UMC has its own scientific and clinical base with a capacity of 856 inpatient beds and 500 outpatient examinations per shift. It unites three innovative medical centers: the Republican Diagnostic Center, the National Scientific Center for Maternal and Child Health and the National research cardiosurgery center. All of them are located in close proximity to each other, have their own staff of specialists, equipment and technical base, which is a modern medical, diagnostic, inpatient, outpatient, preventive healthcare organization. Research centers are accredited according to international JCI standards. The UMC has 80 intensive care beds, 80 cardiology beds and 50 endocrinology beds. UMC has its own CT and MRI scanners, Positron emission tomography. UMC is part of Nazarbayev University's integrated academic healthcare system. UMC has a capacity of >12,000 outpatient visits per year, of which 65-75% are patients with T2D and CVD.

UMC is accredited as a subject of scientific and scientific-technical activities by the Ministry of Education and Science of Kazakhstan (Certificate MK #000058 dated 09.03.2021).

UMC has international partners: Korea University Anam Hospital, Tel-Aviv Sourasky medical center at Tel-Aviv University (Israel), Pittsburg University Medical center (Pittsburgh, USA), University College London, etc. The applicant has official international access to electronic databases (ClarivateAnalytics, WebOfScience, Elsevier, Scopus, ScienceDirect, Springer Nature, SpringerLink, BMJ, BMJUpdate, CochraneLibrary, PubMed, MedLine, Medscape, etc.).

**2)** **Astana Medical University** (Astana, Kazakhstan) (hereafter AMU) [https://amu.edu.kz/en/about-university/] is a major educational, scientific and medical university, has a high reputation in the field of higher medical education. The educational process includes university (bachelor’s degree course, internship) and post-graduate education (residency, magistracy, PhD-doctorate), retraining and advanced training of specialists in the basic educational programs. The university has seven educational and laboratory buildings, six student dormitories for 2500 places. The university has a dissertation council to defend the dissertations of PhD learners.
The university has seven clinical hospitals in Astana. The City Clinical Multidisciplinary Hospital No. 2, which has 500 beds, including 60 therapeutic beds, will participate in this study. [https://www.astanaclinic.kz/index.php/ru/o-nas/o-meditsinskoj-organizatsii]

AMU cooperates with Association of Medical Education in Europe, Association of Medical Schools in Europe, World Health Organization (World Directory of Medical Schools, WHO), International Directory of Universities of UNESCO (International Handbook of Universities, UNESCO).

**3) Genom and personalized medicine laboratory, NLA and NGO "Eurasian Society of Personalized Medicine" (Nazarbayev University)** [https://nla.nu.edu.kz/en/lgpm] will cooperate with the project. The Lab has the following scientific directions: genomic and multimomial studies in biomedicine, genetic studies of multifactorial diseases (oncological, cardiovascular diseases, etc.), genetic of tuberculosis, genetic architecture of Kazakh people, applied genetics.

**4) Nazarbayev University School of Medicine** [https://nusom.nu.edu.kz/] was established to improve the quality of medical education in the Republic of Kazakhstan and to contribute to the transformation of the health care system required to meet the challenges of the health care reform of the country. It will offer the Republic’s first U.S.-style curriculum, taught in English by a diverse faculty of clinicians and biomedical scientists recruited from around the globe.

**Training for researchers**

Every researcher of the team will a great own experience to implement and conduct the scientific investigation which is devoted to a comparative clinical trial.

Manage clinical research project, to recruit severe patients, to make their healthy providing by totally different treatment methods (pharmacological, surgical and diet), follow-up for 24 weeks, use evidence-based medicine principles to publish the clinical results in five of Q1-3 Clarivate-journasl is a challenge for every one of this team. During the study period, young scientists will participate in the proposed project within their Internal Medicine and Surgery residency, bachelor, master, and PhD programs. 5 postdocs (PhD degree holder), 3 PhD students (or master’s degree holder), and 3 master students will be involved to this project***.***

The results of the study will be reported at international conferences and published in journals. During the study, young researchers will be able get knowledge and gain skills from all methods proposed to use in this project, math statistical processing of clinical data, make analysis and interpretation of the results.

**VII. Expected results:**

In an observational epidemiological study and a prospective randomized comparative controlled clinical trial, results will be obtained on the patterns of influence of external transit rhythms of space objects on personalized biorhythms of the patients with T2D on the development in their of MI. For the first time, the relationship pattern between the external transit rhythms of space objects and the internal personalized biorhythms of patients with T2D when they develop MI will be investigated.

For the first time, a mathematical model for predicting the occurrence of MI in patients with T2D will be developed for personalized chrono-prophylaxis of MI. For the first time, the results of the clinical effectiveness of a mathematical model for predicting the occurrence of MI in patients with T2D will be presented for the purpose of personalized chrono-prophylaxis.

**As a result of the research carried out, the following will be published:**

| - at least three articles and/or reviews in peer-reviewed scientific publications indexed in the ScienceCitationIndexExpanded of the WebOfScience database and/or having a CiteScore percentile in the Scopus of at least 50;  - at least 1 patent for an invention (including a positive decision on it); |
| --- |
| - or at least two articles and/or reviews in peer-reviewed scientific publications indexed in ScienceCitationIndexExpanded of the WebOfScience database and/or having a CiteScore percentile in the Scopus of at least 50, at least 1 patent included in the DerwentInnovationsIndex (WebOfScience);  - as well as at least one article or review in a peer-reviewed foreign or domestic publication recommended by the KOKSNVO; |
| - or at least two articles and/or reviews in peer-reviewed scientific publications indexed in ScienceCitationIndexExpanded and included in the 1-2 quartile by impact factor in the WebOfScience database and/or having a CiteScore percentile in the Scopus of at least 65;  - as well as at least one article or review in a peer-reviewed foreign or domestic publication recommended by the KOKSNVO; |
| - or at least one article or review in a peer-reviewed scientific publication included in the 1-2 quartile of the impact factor in the WebOfScience and/or having a CiteScore percentile in the Scopus of at least 65, and at least 1 patent included in the DerwentInnovationsIndex database (WebOfScience);  - as well as at least one article or review in a peer-reviewed foreign or domestic publication recommended by the KOKSNVO; |
| - or at least one article or review in a peer-reviewed scientific publication indexed in the ScienceCitationIndexExpanded and included in the 1st quartile by impact factor in the WebOfScience and/or having a CiteScore percentile in the Scopus of at least 80;  - at least 1 patent for an invention (including a positive decision on it). |

One practical guide.

At least one oral presentation at key academic international conferences.

Applicability of the study results. The results of this project can be effectively used in clinical practice, internal medicine, endocrinology, cardiology, surgery, as well as in primary, secondary and tertiary prevention, also for open private sector.

**Target consumers of the project**

1. Endocrinology, cardiology, bariatric surgeon, diet health care providers and researchers
2. Obese patients with T2D and AH including in drug-intolerance stages
3. Health policy providers.

**VIII. Risk/benefit assessment. Risk level**.

***Risks***. Corresponding to the clinical condition of patients and the treatment interventions being carried out (average).

The safety of surgical treatment is ensured by adequate preoperative examinations, clear indications for bariatric surgery, and the high qualifications of surgical researchers. The goal of this method is to restrict the intake of all types of nutrients (proteins, fats, carbohydrates) through anatomical modification of the gastrointestinal tract, resulting in a negative energy balance (the ratio of energy intake to energy expenditure), gradual weight loss, and a beneficial effect on the course of concomitant diseases..

The safety of pharmacological treatment is ensured by the principles of Pharmacovigilance, namely, monitoring the use of drugs to identify adverse effects and side effects, "scientific and practical activities to identify, evaluate, interpret, and prevent adverse effects or any other drug-related problem," and the high qualifications of endocrinology researchers. Continuous monitoring, recognition, and response to potential or actual problems arising in response to the use of a pharmacological agent.

The safety of the dietary weight loss method (Analytical Detoxification (ANADETO) for the treatment of patients with type 2 diabetes and hypertension) is ensured by timely correction of endogenous metabolic toxicity and psychosocial support for the patient during weight loss. The method is based on the activation of endogenous lipolysis and aims to utilize "old lipids" under the control of endogenous metabolic toxicity..

Nazarbayev University provides laboratory tests to detect TL in patients before and after weight loss therapy..

1. ***Potential risk.*** Given that patients will be recruited for the study as they apply, blood samples for genetic testing will be collected at different times depending on the start of treatment. Blood samples will be transported and stored at the Institute of Genomic Medicine for telomere length determination. Samples will be tested as the special reagent is purchased. Therefore, the risk lies in ensuring proper storage of the collected samples. The Institute of Genomic Medicine has the necessary equipment to ensure this.

2. ***Risk protection.*** Out-of-town patients will have the opportunity to undergo testing 24 weeks after the start of treatment at their place of residence. In this case, storage and transportation of blood samples to the Institute of Genomic Medicine will be provided. The cost of shipping the blood samples is included in the cost of transportation.

3***. Potential benefit to the participant***.

Patients participating in the study will have the opportunity to undergo a full examination, including costly testing: genomic testing, body composition analysis, free consultations, and patient management for 24 weeks. Patients will have the opportunity to recover from their chronic diseases.

5. ***Alternatives for participants***. Patients can receive traditional pathogenetic drug treatment, including under the guaranteed volume of medical care/mandatory health insurance. Patients will be given the opportunity to choose alternative treatments available at the Foundation's departments.

**IX. Study Participant Identification, Recruitment, and Consent**

1. The study will involve 150 patients aged 30 to 60 years. This age range was chosen to ensure high-quality genetic testing. There is evidence that the importance of telomere length increases with aging. Telomere length changes between 30 and 60 years provide an opportunity to more accurately study lifespan. Patients will be recruited only with written informed consent. Patients will be assigned to groups using a traditional randomization method.

***2. Participant identification and recruitment methods.*** To recruit patients for the comparison groups, consultation meetings will be held with primary care physicians. At these meetings, the terms and benefits for patients participating in this study will be explained in detail. A patient database is available at the Regional Clinical Center and the Kazakhstan Society of Bariatric and Metabolic Surgeons for recruitment into the 3 groups. Participation in the study will be offered based on inclusion and exclusion criteria.

***3. Consent process.*** Before the study begins, the study director/co-director will conduct informational interviews with patients, describing the terms of participation, benefits, and potential risks. During control group recruitment, informed consent will be obtained by senior investigators. The senior investigators will maintain records of signed informed consents.

4***. Participant status.*** Competent patients aged 30 to 60 years will be eligible to participate in the study. Participants who do not sign informed consent will not be included in the study.

5. ***Understanding.*** All researchers have a legal and ethical obligation to ensure that prospective subjects or their representatives have sufficient knowledge and understanding of the elements of informed consent to enable them to make an informed and informed decision whether or not to participate, or to consent to participation in the study. In this section, describe how it will be determined that the subject or their legally authorized representative has understood the information presented. This section should clearly reflect an adequate plan to ensure an acceptable level of understanding before consent is obtained. If children and/or incompetent adults will participate, this section should also include a specific plan for assessing understanding during consent.

6. ***Consent forms.*** The informed consent form will be approved by the local ethics committee and will have the medical organization's footers.

***7. Documentation of consent.*** The principal investigator is responsible for obtaining and documenting the informed consent from all subjects. The documentation and storage of the informed consent will be carried out by the researcher and will be included in the patient's medical record.

8***. Cost of patient participation.*** None. Patient testing, including expensive ones (genetic, body composition analysis), as well as the weight loss program (medication, MGS, and ANADETO) will be covered by a research grant.

***9. Participation fee.*** None.

**X. References**

[1] OshakbayevKP KA, PonomarevIO , GazaliyevaMA , Dukenbayeva BA, Oshakbayev P, Zhumabekova BK. , Kaliyeva Sh., Shakeyev K. Weight loss program in patients with atherosclerosis: a randomised clinical trial. 13-F. Global Journal of Medical Research: Global Journal of Medical Research; 2013:51-60.

[2] Oshakbayev K, Dukenbayeva B, Togizbayeva G, Durmanova A, Gazaliyeva M, Sabir A, et al. Weight loss technology for people with treated type 2 diabetes: a randomized controlled trial. Nutrition & Metabolism 2017;14.

[3] Oshakbayev KP, Alibek K, Ponomarev IO, Uderbayev NN, Dukenbayeva BA. Weight change therapy as a potential treatment for end-stage ovarian carcinoma. Am J Case Rep 2014;15:203-11.

[4] Rizza W, Veronese N, Fontana L. What are the roles of calorie restriction and diet quality in promoting healthy longevity? Ageing Research Reviews 2014;13:38-45.

[5] Salvestrini V, Sell C, Lorenzini A. Obesity May Accelerate the Aging Process. Frontiers in Endocrinology 2019;10.

[6] Athanasakis K, Prodromiadou E, Papazafiropoulou A, Koutsovasilis A, Driva S, Ziori M, et al. Twenty-year trends in the prescription costs of Type 2 diabetes: Real world data and empirical analysis in Greece. Diabetes Research and Clinical Practice 2020;162.

[7] Larsson SC, Burgess S. Causal role of high body mass index in multiple chronic diseases: a systematic review and meta-analysis of Mendelian randomization studies. Bmc Medicine 2021;19(1).

[8] Hashmi SFA, Dasti MA, Baloch GH, Zaidi SI, Bukhari S, Baloch ZAQ. FREQUENCY OF CARDIAC COMPLICATIONS IN PATIENTS WITH TYPE 2 DIABETES MELLITUS AT TERTIARY CARE HOSPITAL. Indo American Journal of Pharmaceutical Sciences 2017;4(2):296-301.

[9] Swoboda PP, McDiarmid AK, Erhayiem B, Haaf P, Kidambi A, Fent GJ, et al. A Novel and Practical Screening Tool for the Detection of Silent Myocardial Infarction in Patients With Type 2 Diabetes. Journal of Clinical Endocrinology & Metabolism 2016;101(9):3316-23.

[10] Arenja N, Mueller C, Ehl NF, Brinkert M, Roost K, Reichlin T, et al. Prevalence, Extent, and Independent Predictors of Silent Myocardial Infarction. American Journal of Medicine 2013;126(6):515-22.

[11] Sultan A, Perriard F, Macioce V, Mariano-Goulart D, Boegner C, Daures JP, et al. Evolution of silent myocardial ischaemia prevalence and cardiovascular disease risk factor management in Type 2 diabetes over a 10-year period: an observational study. Diabetic Medicine 2017;34(9):1244-51.

[12] Sarria-Santamera A, Orazumbekova B, Maulenkul T, Gaipov A, Atageldiyeva K. The Identification of Diabetes Mellitus Subtypes Applying Cluster Analysis Techniques: A Systematic Review. International Journal of Environmental Research and Public Health 2020;17(24).

[13] Liu J, Lavie CJ, Park YMM, Bagiella E. Geographic variation and trends in prevalence of obesity among US adolescents, 2016-2021. Public Health 2023;223:128-30.

[14] Galiyeva D, Gusmanov A, Sakko Y, Issanov A, Atageldiyeva K, Kadyrzhanuly K, et al. Epidemiology of diabetes in Kazakhstan: data from unified nationwide electronic healthcare system 2014-2019. Diabetologia 2021;64(SUPPL 1):138-.

[15] Orazumbekova B, Issanov A, Atageldiyeva K, Berkinbayev S, Junusbekova G, Danyarova L, et al. Prevalence of Impaired Fasting Glucose and Type 2 Diabetes in Kazakhstan: Findings From Large Study. Frontiers in Public Health 2022;10.

[16] Aminian A, Wilson R, Al-Kurd A, Tu C, Milinovich A, Kroh M, et al. Association of Bariatric Surgery With Cancer Risk and Mortality in Adults With Obesity. JAMA; June  3,  2022.

[17] Tahrani A, Morton J. Benefits of weight loss of 10% or more in patients with overweight or obesity: A review. Obesity 2022;30(4):802-40.

[18] Horn DB, Almandoz JP, Look M. What is clinically relevant weight loss for your patients and how can it be achieved? A narrative review. Postgraduate Medicine 2022.

[19] Henteleff HJ, Birch DW, Hallowell PT, Evidence Based Reviews Surg G. Cost-effectiveness of bariatric surgery for severely obese adults with diabetes. Canadian Journal of Surgery 2013;56(5):353-5.

[20] Raj S, Williams EM, Davis MJ, Abu-Ghname A, Luu BC, Buchanan EP. Cost-effectiveness of Multidisciplinary Care in Plastic Surgery A Systematic Review. Annals of Plastic Surgery 2021;87(2):206-10.

[21] Bowman K, Atkins JL, Delgado J, Kos K, Kuchel GA, Ble A, et al. Central adiposity and the overweight risk paradox in aging: follow-up of 130,473 UK Biobank participants. American Journal of Clinical Nutrition 2017;106(1):130-5.

[22] Mueller MJ, Geisler C. From the past to future: from energy expenditure to energy intake to energy expenditure. European Journal of Clinical Nutrition 2017;71(3):358-64.

[23] Haywood CJ, Prendergast LA, Lim R, Lappas M, Lim WK, Proietto J. Obesity in older adults: Effect of degree of weight loss on cardiovascular markers and medications. Clinical Obesity 2019;9(4).

[24] Most J, Redman LM. Impact of calorie restriction on energy metabolism in humans. Experimental Gerontology 2020;133.

[25] Shammas MA. Telomeres, lifestyle, cancer, and aging. Current Opinion in Clinical Nutrition and Metabolic Care 2011;14(1):28-34.

[26] Verma AK, Singh P, Al-Saeed FA, Ahmed AE, Kumar S, Kumar A, et al. Unravelling the role of telomere shortening with ageing and their potential association with diabetes, cancer, and related lifestyle factors. Tissue & Cell 2022;79.

[27] Raftopoulou C, Paltoglou G, Charmandari E. Association between Telomere Length and Pediatric Obesity: A Systematic Review. Nutrients 2022;14(6).

[28] KrishnaKuchipudi GS, Prabhu M. Study of Association of Leptin and Leucocyte Telomere Length with Body Mass Index in Adult Indian Population a One Year Cross Sectional Study. The Journal of the Association of Physicians of India 2022;70(4):11-2.

[29] Whittemore K, Vera E, Martínez-Nevado E, Sanpera C, Blasco MA. Telomere shortening rate predicts species life span. Proceedings of the National Academy of Sciences of the United States of America 2019;116(30):15122-7.

[30] Cheng FF, Luk AO, Wu HJ, Lim CKP, Carroll L, Tam CHT, et al. Shortened relative leukocyte telomere length is associated with all-cause mortality in type 2 diabetes-analysis from the Hong Kong Diabetes Register. Diabetes Research and Clinical Practice 2021;173.

[31] Testa R, Olivieri F, Sirolla C, Spazzafumo L, Rippo MR, Marra M, et al. Leukocyte telomere length is associated with complications of Type 2 diabetes mellitus. Diabetic Medicine 2011;28(11):1388-94.

[32] Gielen M, Hageman GJ, Antoniou EE, Nordfjall K, Mangino M, Balasubramanyam M, et al. Body mass index is negatively associated with telomere length: a collaborative cross-sectional meta-analysis of 87 observational studies. American Journal of Clinical Nutrition 2018;108(3):453-75.

[33] Oshakbayev K. AZ, Zhumabekova B.K., Gazaliyeva M.A., Dukenbayeva B.A.,Oshakbayev P. Prime risk factor of cardiovascular diseases and Weight loss program in the Kazakh population: a Panel study. Journal of Medical and Biological Sciences (Scientific Journals International) 2012;5(1):8.

[34] Moreira VDA, Gonzalez MDM, Pino AVL, Mena DST, Villacres VZC, Benavides KMR, et al. Differential characteristics of empagliflozin in the treatment of diabetes mellitus. Revista Latinoamericana De Hipertension 2021;16(2):163-+.

[35] Oshakbayev KP, Seidaliyeva AP, Togizbayeva GI, Gazaliyeva MA, Durmanova AK, Idrisov AS, et al. Clinical management with weight loss therapy in patients with Type 2 diabetes: a randomised clinical trial. Diabetic Medicine 2016;33:32-.

[36] Oshakbayev K, Bimbetov B, Manekenova K, Bedelbayeva G, Mustafin K, Dukenbayeva B. Severe nonalcoholic steatohepatitis and type 2 diabetes: liver histology after weight loss therapy in a randomized clinical trial. Current Medical Research and Opinion 2019;35(1):157-65.

[37] Ospanov O, Akilzhanova A, Bekmurzinova F, Fursov R. TELOMERE LENGTH AS A MARKER OF LIFE EXPECTANCY IS ELONGATED AFTER GASTRIC BYPASS OF PATIENTS WITH METABOLIC SYNDROME Basic science and research in bariatric surgery. Obesity Surgery 2019;29:461-.

[38] Ospanov O, Akilzhanova A, Buchwald JN, Fursov A, Bekmurzinova F, Rakhimova S, et al. Stapleless vs Stapled Gastric Bypass vs Hypocaloric Diet: a Three-Arm Randomized Controlled Trial of Body Mass Evolution with Secondary Outcomes for Telomere Length and Metabolic Syndrome Changes. Obesity Surgery 2021;31(7):3165-76.

[39] Serhal S, Mitchell B, Krass I, Emmerton L, Bereznicki B, Bereznicki L, et al. Rethinking the gold standard - The feasibility of randomized controlled trials within health services effectiveness research. Research in Social & Administrative Pharmacy 2022;18(9):3656-68.

[40] Powell K, Prasad V. Common misconceptions of randomized controlled trials in oncology. European Journal of Clinical Investigation 2022;52(11).

[41] Grossman J, Mackenzie FJ. The randomized controlled trial - gold standard, or merely standard? Perspectives in Biology and Medicine 2005;48(4):516-34.

[42] Christ TW. Scientific-Based Research and Randomized Controlled Trials, the "Gold" Standard? Alternative Paradigms and Mixed Methodologies. Qualitative Inquiry 2014;20(1):72-80.

[43] Hutton B, Fergusson D. Changes in body weight and serum lipid profile in obese patients treated with orlistat in addition to a hypocaloric diet: a systematic review of randomized clinical trials. American Journal of Clinical Nutrition 2004;80(6):1461-8.

[44] Hickey GL, Grant SW, Dunning J, Siepe A. Statistical primer: sample size and power calculations-why, when and how? European Journal of Cardio-Thoracic Surgery 2018;54(1):4-9.

[45] Zhong P, Zeng H, Huang MC, Fu WB, Chen ZX. Efficacy and safety of once-weekly semaglutide in adults with overweight or obesity: a meta-analysis. Endocrine 2022;75(3):718-24.

[46] Lingvay I, Capehorn MS, Catarig AM, Johansen P, Lawson J, Sandberg A, et al. Efficacy of Once-Weekly Semaglutide vs Empagliflozin Added to Metformin in Type 2 Diabetes: Patient-Level Meta-analysis. Journal of Clinical Endocrinology & Metabolism 2020;105(12).

[47] Buse JB, Wexler DJ, Tsapas A, Rossing P, Mingrone G, Mathieu C, et al. 2019 Update to: Management of Hyperglycemia in Type 2 Diabetes, 2018. A Consensus Report by the American Diabetes Association (ADA) and the European Association for the Study of Diabetes (EASD). Diabetes Care 2020;43(2):487-93.

[48] Davies MJ, Aroda VR, Collins BS, Gabbay RA, Green J, Maruthur NM, et al. Management of Hyperglycemia in Type 2 Diabetes, 2022. A Consensus Report by the American Diabetes Association (ADA) and the European Association for the Study of Diabetes (EASD). Diabetes Care 2022;45(11):2753-86.

[49] Gandhi GY, Mooradian AD. Management of Hyperglycemia in Older Adults with Type 2 Diabetes. Drugs & Aging 2022;39(1):39-58.

[50] Matar M, Mahfouz MF, Salama TMS. Minigastric bypass compared with Roux-en-Y gastric bypass after failed vertical banding gastroplasty: a retrospective cohort of 100 patients. Egyptian Journal of Surgery 2021;40(4):1476-80.

[51] Navarrete Aulestia S, Leyba JL, Navarrete Llopis S, Pulgar V. One Anastomosis Gastric Bypass/Minigastric Bypass in Patients with BMI < 35 kg/m<SUP>2</SUP> and Type 2 Diabetes Mellitus: Preliminary Report. Obesity Surgery 2019;29(12):3987-91.

[52] Oshakbayev K, Dukenbayeva B, Otarbayev N, Togizbayeva G, Tabynbayev N, Gazaliyeva M, et al. Weight loss therapy for clinical management of patients with some atherosclerotic diseases: a randomized clinical trial. Nutrition Journal 2015;14:9.

[53] Oshakbayev K, Bimbetov B, Manekenova K, Bedelbayeva G, Mustafin K, Dukenbayeva B. Severe nonalcoholic steatohepatitis and type 2 diabetes: liver histology after weight loss therapy in a randomized clinical trial. Current medical research and opinion 2018:1-24.

[54] Sodhi M, Rezaeianzadeh R, Kezouh A, Etminan M. Risk of Gastrointestinal Adverse Events Associated With Glucagon-Like Peptide-1 Receptor Agonists for Weight Loss. Jama-Journal of the American Medical Association 2023.

[55] ElSayed N, Aleppo GR, Aroda VR, Bannuru RM, Brown F, Bruemmer DS, et al. 2. Classification and diagnosis of diabetes: Standards of Care in Diabetes-2023 (vol 46, pg S19, 2023). Diabetes Care 2023;46(5):1106-.

[56] Fang H, Berg E, Cheng X, Shen W. How to best assess abdominal obesity. Current Opinion in Clinical Nutrition and Metabolic Care 2018;21(5):360-5.

[57] Cawthon RM. Telomere length measurement by a novel monochrome multiplex quantitative PCR method. Nucleic Acids Research 2009;37(3).

| **Client:**  "Science Committee of the Ministry of Science and Higher Education of the Republic of Kazakhstan"  Astana, Mangilik El avenue, 8  **Contractor:**  Corporate Foundation "University Medical Center",  Astana, Kerey Zhanibek Kh. Street, 5/1  **Responsible Contractor:**  Project Supervisor  ____________ prof. K.P. Oshakbayev |  |
| --- | --- |

**Table.** Implementation work plan

| #  # | Name of tasks  and measures for their implementation | Implementation date | | Expected results of the project (in terms of tasks and activities), the form of completion |  |  |
| --- | --- | --- | --- | --- | --- | --- |
|  |  | Start  (month) | End  (month) |  |  |  |
| **2024 year** | | | | | |  |
|  | Development and approval of the research protocol, quality of life questionnaire, criteria for assessing cost effectiveness. | January | February | Protocol, questionnaire. |  |  |
|  | Approval of the study protocol, quality of life questionnaire, and criteria for assessing economic effectiveness in the local ethics committee. | January | February | Ethical committee conclusion. |  |  |
|  | Registration of the study on ClinicalTrials.gov | March | April | Trial registration number on ClinicalTrials.gov |  |  |
|  | Recruitment of patients, signing the informed consent form | February | December | Database of patients with T2D and AH with obesity |  |  |
|  | Laboratory blood sampling before surgery. | February | December | Laboratory database of patients with T2D and AH with obesity |  |  |
|  | TL blood collection before intervention. | February | December | Database of TL patients with T2D and AH with obesity |  |  |
|  | Assessment of quality of life before intervention | February | December | Database of quality of life of patients with T2D and AH with obesity |  |  |
|  | Treatment period, monitoring, observation. | February | December | Database of treatment and diagnostic data for patients with T2D and AH with obesity. |  |  |
|  | Patent search and literature review | July | December | Results of patent search and literature review |  |  |
|  | Preparation and submission for publication of the 1st article in Q1-3 of the Web of Science database and/or having a CiteScore percentile in the Scopus database of at least 50. | October | December | The first publication in Q1-3 of the Web of Science database and/or having a CiteScore percentile in the Scopus database of at least 50 or submitted number. |  |  |
| **2025 year** | | | | | |  |
|  | Recruitment of patients, signing the informed consent form | January | December | Database of patients with T2D and AH with obesity |  |  |
|  | Laboratory blood sampling before surgery. | January | December | Laboratory database of patients with T2D and AH with obesity |  |  |
|  | TL blood collection before intervention. | January | December | Database of TL patients with T2D and AH with obesity |  |  |
|  | Assessment of quality of life before intervention | January | December | Database of quality of life of patients with T2D and AH with obesity |  |  |
|  | Laboratory blood sampling after surgery. | January | December | Laboratory database of patients with T2D and AH with obesity |  |  |
|  | TL blood collection after intervention. | January | December | Database of TL patients with T2D and AH with obesity |  |  |
|  | Assessment of quality of life after intervention | January | December | Database of quality of life of patients with T2D and AH with obesity |  |  |
|  | Treatment period, monitoring, observation. | January | December | Database of treatment and diagnostic data for patients with T2D and AH with obesity. |  |  |
|  | Intermediate statistical analysis process. | August | December | Intermediate analysis. |  |  |
|  | Preparation and submission for publication of the 2nd article in Q1-3 of the Web of Science database and/or having a CiteScore percentile in the Scopus database of at least 50. | October | December | The second publication in Q1-3 of the Web of Science database and/or having a CiteScore percentile in the Scopus database of at least 50 or submitted number. |  |  |
|  | Application for an Invention Patent | August | December | Invention patent application submission number |  |  |
| **2026 year** | | | | | | Patent search and literature review |
|  | Treatment period, monitoring, observation. | January | April | Database of treatment and diagnostic data for patients with T2D and AH with obesity. |  |  |
|  | Assessment of side effects after interventions | January | May | Database of side effects after interventions in patients with T2D and AH with obesity. |  |  |
|  | Assessment of quality of life after intervention | January | May | Database of quality of life of patients with T2D and AH with obesity |  |  |
|  | Evaluation of cost effectiveness after interventions | January | May | Database of cost effectiveness analysis after interventions in patients with T2D and AH with obesity |  |  |
|  | Final process of statistical analysis. | June | August | Final statistical analysis. |  |  |
|  | Study closure on ClinicalTrials.gov | October | November | Study closure on ClinicalTrials.gov |  |  |
|  | Patent for invention. | August | December | Patent for invention or positive decision |  |  |
|  | Preparation and submission for publication of the 3rd article in Q1-3 of the Web of Science database and/or having a CiteScore percentile in the Scopus database of at least 50. | September | December | The 3rd publication in Q1-3 of the Web of Science database and/or having a CiteScore percentile in the Scopus database of at least 50 or submitted number. |  |  |
|  | Evaluation of results | September | October | Outcome Evaluation Data |  |  |
|  | Final assessment and results | November | December | Final results |  |  |
